# Beyond the Whole-Genome Duplication: Phylogenetic Evidence for an Ancient Interspecies Hybridization in the Baker's Yeast Lineage

**DOI:** 10.1371/journal.pbio.1002220

**Published:** 2015-08-07

**Authors:** Marina Marcet-Houben, Toni Gabaldón

**Affiliations:** 1 Centre for Genomic Regulation (CRG), Barcelona, Spain; 2 Universitat Pompeu Fabra (UPF), Barcelona, Spain; 3 Institució Catalana de Recerca i Estudis Avançats (ICREA), Barcelona, Spain; University of Bath, UNITED KINGDOM

## Abstract

Whole-genome duplications have shaped the genomes of several vertebrate, plant, and fungal lineages. Earlier studies have focused on establishing when these events occurred and on elucidating their functional and evolutionary consequences, but we still lack sufficient understanding of how genome duplications first originated. We used phylogenomics to study the ancient genome duplication occurred in the yeast *Saccharomyces cerevisiae* lineage and found compelling evidence for the existence of a contemporaneous interspecies hybridization. We propose that the genome doubling was a direct consequence of this hybridization and that it served to provide stability to the recently formed allopolyploid. This scenario provides a mechanism for the origin of this ancient duplication and the lineage that originated from it and brings a new perspective to the interpretation of the origin and consequences of whole-genome duplications.

## Abstract 2

The ancient whole-genome duplication in the *Saccharomyces cerevisiae* lineage has been a paradigm for the study of genome duplications in eukaryotes. This article presents evidence for the existence of an interspecies hybridization event shortly before the duplication, with deep implications for the functional and evolutionary consequences of this genome doubling.

## Author Summary

Genome duplication is a major evolutionary process that has shaped the genomes of several eukaryotic lineages including vertebrates, plants, and fungi. The sequencing of the baker's yeast *Saccharomyces cerevisiae* in the 1990s revealed the presence of conserved blocks of duplicated genes, indicating an ancestral duplication of the entire genome. Subsequent work has clarified when this event occurred and what genomic rearrangements followed, but the underlying mechanistic origin of such a large-scale event remains poorly understood. Here we used a large-scale phylogenetic approach to examine the individual evolutionary histories of all yeast genes and assessed the time at which each duplication occurred. This survey revealed evidence for an ancient hybridization event between two ancestral species in the lineage in which the whole-genome duplication had occurred. We further characterize this hybridization event and the properties of the putative parental species. We propose that the whole-genome duplication was a direct consequence of this hybridization, providing a means by which the initially sterile hybrid could regain fertility. This scenario provides a mechanistic understanding of the origin of the ancient yeast whole-genome duplication and brings a radically different perspective on the interpretation of the origin and evolutionary consequences of whole-genome duplications in eukaryotic lineages.

## Introduction

**Fig 1:**
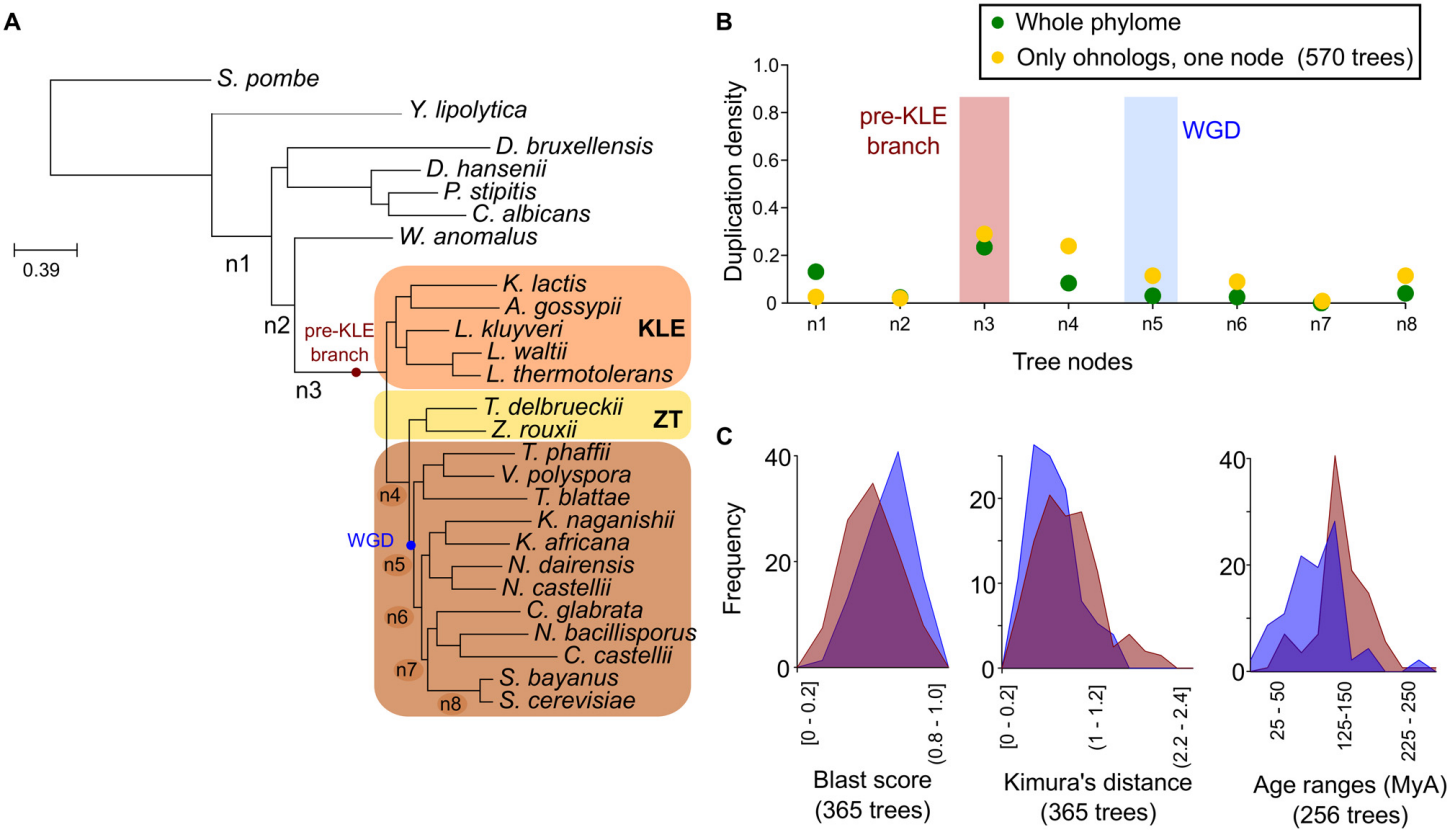
Evidence of a duplication peak pre-dating the WGD. (A) Evolutionary relationships of the analysed species. The tree was built using a maximum likelihood approach on a concatenated alignment of 516 widespread orthologs. All branches had maximal bootstrap support (100%). The WGD and the pre-KLE (*Kluyveromyces*, *Lachancea*, and *Eremothecium*) branch are marked with coloured circles. Branches in the lineage leading from *S*. *cerevisiae* to the root are numbered from more ancestral (n1) to more recent (n8). (B) Duplication densities (duplications per gene per branch) calculated for each annotated branch, either using the entire set of gene trees (green dots) or only the ohnologs (yellow dots). (C) Sequence divergence between yeast sequences belonging to two populations: duplication mapped at the WGD branch (blue) and duplication mapped at the pre-KLE branch (red). Graphs represent frequencies of normalized blast scores, Kimura distances, and estimated divergence age, respectively. Normalized blast score is the result of dividing the blast score obtained when aligning the seed yeast protein to the ohnolog pair by the blast score obtained from aligning the seed yeast protein to itself. The Kimura distance between the two sequences was calculated using protdist as implemented in the phylip package after aligning the two sequences. PL-R8s [14] was used to assess the divergence times in individual trees that contained two ohnologous genes. Data on which this figure is based are provided in S1 Data.

Ancient whole-genome duplications (WGDs) are major evolutionary events that have impacted several eukaryotic lineages, including plants, animals, and fungi [1]. Among plants, ancestral WGDs have been identified in monocots and core eudicots [2], and more recent events are apparent in many lineages such as *Arabidopsis*, maize, and soybean [3–5]. In vertebrates, the existence of two ancestral WGDs (but also more recent ones in teleost fishes and frogs) has been proposed [2]. Earlier work has focused on establishing the periods at which these events occurred [6,7] and on assessing the functional and evolutionary aftermath of the doubling of the entire genetic complement [8]. However, we still do not fully understand what initially triggered these events. Perhaps the best-studied WGD is the one affecting an ancestor of the baker's yeast *Saccharomyces cerevisiae*, an event supported by the finding of numerous blocks of paralogs with conserved synteny [7,9]. It is now established that this event occurred just before the separation of *Vanderwaltozyma polyspora* from the *S*. *cerevisiae* lineage, originating a clade of post-WGD species (Fig 1A) [10]. In addition, it has been shown that the genome doubling was followed by extensive genome rearrangements and rampant gene loss that have since shaped these species' genomes, resulting in only a minor fraction of the WGD-derived paralogs (ohnologs) being retained [11,12]. Based on the high level of synteny found between reconstructed ancestrally duplicated gene blocks, it has been proposed that the yeast WGD has its origin in an autopolyploidization event [11]. This proposition has important implications with respect to the possible initial selective advantages that played a role after the polyploidization event. Polyploidy has been considered to promote evolutionary innovation because it facilitates neo- and subfunctionalization and buffers deleterious mutations. However, these mechanisms only provide an advantage after some time has passed and a number of mutations have accumulated. Conversely, simple increase in ploidy has been considered to put barriers to fast adaptation, as it masks beneficial recessive mutations and avoids rapid purging of deleterious mutations. Furthermore, most experimental work comparing populations of different ploidy generally provides support for the superiority of the normal ploidy versus increased ploidies in a given species [13]. Thus, the nature of the initial evolutionary advantage of the yeast WGD remains an open question.

WGDs leave a footprint in the form of cohorts of homologous genes that duplicated in the same period. Phylogenetic analysis of gene families informs on the relative age of duplications [15,16] and hence is a powerful tool to study WGDs. When ancestral duplications are inferred from the genes encoded in a genome and their relative dates are mapped to a reference species tree, ancient WGDs are expected to lead to an accumulation of duplications mapped to the lineage in which the event occurred. Earlier analyses have used such approach to detect ancient duplications in vertebrates [17,18] and plants [19]. However, despite extensive phylogenetic work [20–22], no study has assessed the global phylogenetic congruence of gene duplications and the WGD that occurred in the lineage leading to *S*. *cerevisiae*. Here, we set out to investigate patterns of past duplications in *S*. *cerevisiae* by analysing genome-wide sets of gene phylogenies (i.e., phylomes).

## Results and Discussion

### Gene Phylogenies Reveal Waves of Ancestral Duplications

We based our analyses on a set of 26 completely sequenced genomes, for which we reconstructed a reference species phylogeny based on the alignment concatenation of 516 widespread, single-copy orthologs (See Fig 1A, Materials and Methods). Subsequently, we used the phylomeDB pipeline [23] to reconstruct the evolutionary history of every protein encoded in the *S*. *cerevisiae* genome. These gene family trees were used to detect and date well-supported duplication events, using a phylogeny-based method described elsewhere [16]. In brief, the method exploits the temporal information provided by the branching patterns in a given gene tree: a duplication must be older than the lineages diverging subsequent to it and younger than lineages branching earlier. Using this information, we can map duplications to the reference species tree and compute duplication densities per gene and branch (S1 Fig). Unexpectedly, our analyses revealed the largest duplication peak (0.28 duplications per gene) at the branch preceding the divergence between *Saccharomyces* and a clade containing the genera *Kluyveromyces*, *Lachancea*, and *Eremothecium* (*Ashbya gossypii*) [24], hereafter referred to as KLE (Fig 1B). To assess whether this peak was indeed related to the WGD event, we limited our analysis to those duplications leading to conserved pairs of WGD-ohnologs as defined in the Yeast Gene Order Browser (YGOB) database [25]. Note that YGOB uses a synteny criterion which is independent of the specific gene phylogeny. We found that the pre-KLE duplication peak was more apparent in the subset of duplications leading to conserved pairs of ohnologs, which indicates that this ancestral duplication peak is indeed related to the observed WGD paralogous blocks (Fig 1A and 1B). Of note, not all duplications resulting in pairs of conserved, syntenic ohnologs mapped to the pre-KLE peak (n3). A second accumulation of duplications appeared at the branch preceding the divergence of a clade formed by *Zygosaccharomyces rouxii* and *Torulaspora delbrueckii* (referred to as ZT hereafter) with the post-WGD species (n4). A smaller fraction of duplications mapped to the expected WGD location (n5) or subsequent branches. We assessed the degree of divergence between syntenic ohnologs derived from duplications at the pre-KLE peak and those from duplications at the WGD node, as the two more divergent points of interest, and found that the former had significantly larger divergences (Fig 1C). This supports that gene pairs whose duplications are predicted to be more ancestral by a topological approach are also more divergent at the sequence level. It also indicates that the genes in paralogous blocks may be composed of distinct sets of genes, diverged at different times.

### The Ancestral Duplication Peak Is Robust

To discard the possibility that our unexpected result was artifactual and to understand what may have caused the dispersion of the duplication mappings outside the WGD node, we carefully assessed possible methodological and interpretation pitfalls. First of all, given that the pre-KLE branch is among the longest in our species phylogeny, the ancestral peak could simply indicate a higher number of duplications accumulated over a longer period of time. We thus measured the correlation between duplication densities and branch lengths for the whole phylogeny. While a high correlation was indeed observed when considering all the duplications (r^2^ = 0.92 Pearson), this was not the case when the analysis was restricted to only those duplications leading to syntenic ohnologs (r^2^ = 0.00). We next assessed the effect of using alternative yeast species as a seed in the phylome reconstruction and observed that the use of *Candida glabrata* or *V*. *polyspora* phylomes resulted in similar patterns of duplication densities (see S2 Fig). Another, always contentious point is the use of a reference phylogeny. Although the reconstructed species tree was highly supported and congruent with earlier reconstructions [24], an alternative branching order for the KLE species had been previously presented [26]. This alternative topology suggested that the *Lachancea*, *Kluyveromyces*, and *Eremothecium* are not monophyletic but rather stem out sequentially from the lineage leading to *S*. *cerevisiae* (see S3 Fig). Such organization could potentially affect our results if, for instance, the pre-KLE duplications were found to be partitioned among the new internodes (i.e., branches) created by this topology. To test this, we repeated the analysis using the alternative topology as a reference. Our results show that the underlying topology does not affect the central finding that an apparent duplication peak existed before the divergence of KLE species (S3 Fig). Finally, we tried an alternative method to map the duplication events of ohnologs by using the reconciliation-based algorithm implemented in Notung [27], which rendered similar results (S4 Fig). Thus, a different species topology and a different duplication detection method do not alter the main result that the majority of ohnologs have apparently diverged before the expected WGD.

We next tried to assess the possible effect of stochastic errors or artifacts in the gene trees. We did so by focusing on the trees that contained pairs of conserved ohnologs in *S*. *cerevisiae*. Short sequences tend to be less reliable and more prone to stochastic errors. First, we examined the signal present in subsets of sequences of varying lengths (<500 aa, 500 to 1,000 aa, and >1,000 aa). As seen in (S5 Fig), the three groups of genes consistently provide a very low duplication signal at the WGD, while signals at the two previous branches (pre-ZT and pre-KLE) are much larger.

Secondly, we assessed the robustness of our main result across a range of different methodological approaches for gene tree reconstruction. We tested three different maximum likelihood programs: PhyML [28], RAxML [29], and Fasttree [30]; and one program based on Bayesian inference (BI): Phylobayes [31]. In addition to the best-fitting evolutionary model used in our standard analyses, we used PhyML to test the effect of using two different, more complex models (C20-CAT [32] and Covarion [33]) and a different search heuristic, subtree pruning and regrafting (SPR), instead of the default nearest-neighbour interchange (NNI). Finally, we tested two different support methods in RAxML (rapid bootstrapping and Shimodaira–Hasegawa (SH) support) and in PhyML (approximate likelihood ratio test [aLRT] and bootstrapping). A summary of the different methods can be found in S1 Table. Results of different methods are not directly comparable because different subsets of trees pass the filters for a given procedure (see S6 Fig). However, when a tree passed the filters for any given two methods, the result was highly consistent in most cases (86% overall agreement). Overall, our main result that duplications are apparently older than the expected WGD remained consistent (see S7 Fig). The fraction of ohnolog duplications mapped to the expected WGD node is minimal (<15% in all datasets), while more ancestral duplications are prominent with >50% of the duplications being mapped to the two nodes preceding the WGD (pre-ZT and pre-KLE), although the balance between these two prominent peaks differed between the methods. These differences notwithstanding, the main conclusion of the duplication density analysis is consistent across methods: the majority of ohnologs have inferred duplication ages that predate the expected time of the WGD.

Finally, phylogenetic artifacts such as long-branch attraction (LBA) can produce wrong topologies with high support [34]. It is possible that trees containing paralogs diverging at very unequal rates may have been affected by LBA, misplacing duplications closer to the root. In fact, differential rates among paralogs are expected when processes of neofunctionalization are acting. One way to ascertain whether LBA is affecting the topology is reconstructing the tree with and without the out-groups. In the absence of LBA, the in-group topology is expected to remain stable [34]. We applied this test to the trees containing ohnologs and found that the majority of trees (85%) gave consistent mappings of the duplication of the ohnologs, indicating that the effect of LBA is not widespread and does not significantly affect the duplication mapping. We performed a second test to see whether LBA could explain the observed patterns. For this, we devised sequence simulations in which one of the ohnologs was made to evolve 20 times faster than its paralog. Despite the use of such extreme values, the duplication peak at simulations was detectable at the expected location, and artifactual peaks were significantly smaller and not apparent at the pre-KLE lineage (see Materials and Methods, S8 Fig). Gene conversion among duplicates may result in underestimation of duplication ages, possibly accounting for part of the disappearance of the WGD peak, but not for the presence of the pre-KLE peak. Thus, LBA and gene conversion may have blurred the signal of the WGD peak but cannot account for the prominent pre-KLE peak.

Our results show compelling evidence that a majority of yeast genes defined as ohnologs have diverged before the expected period of the WGD. This overall result holds even though the exact mapping from individual gene trees may vary across methodologies and datasets. The event under study is very ancient, and genes contain a limited amount of information; thus, degradation of the signal is expected. However, stochastic noise would explain a diffusion of the signal but not the existence of a stronger, more ancient duplication peak. We have also shown that distorting processes such as LBA cannot account for the observed patterns. We thus turned to assess other possible biological explanations for our observation.

### Hybridization Accounts for the Observed Phylogenetic Patterns

**Fig 2:**
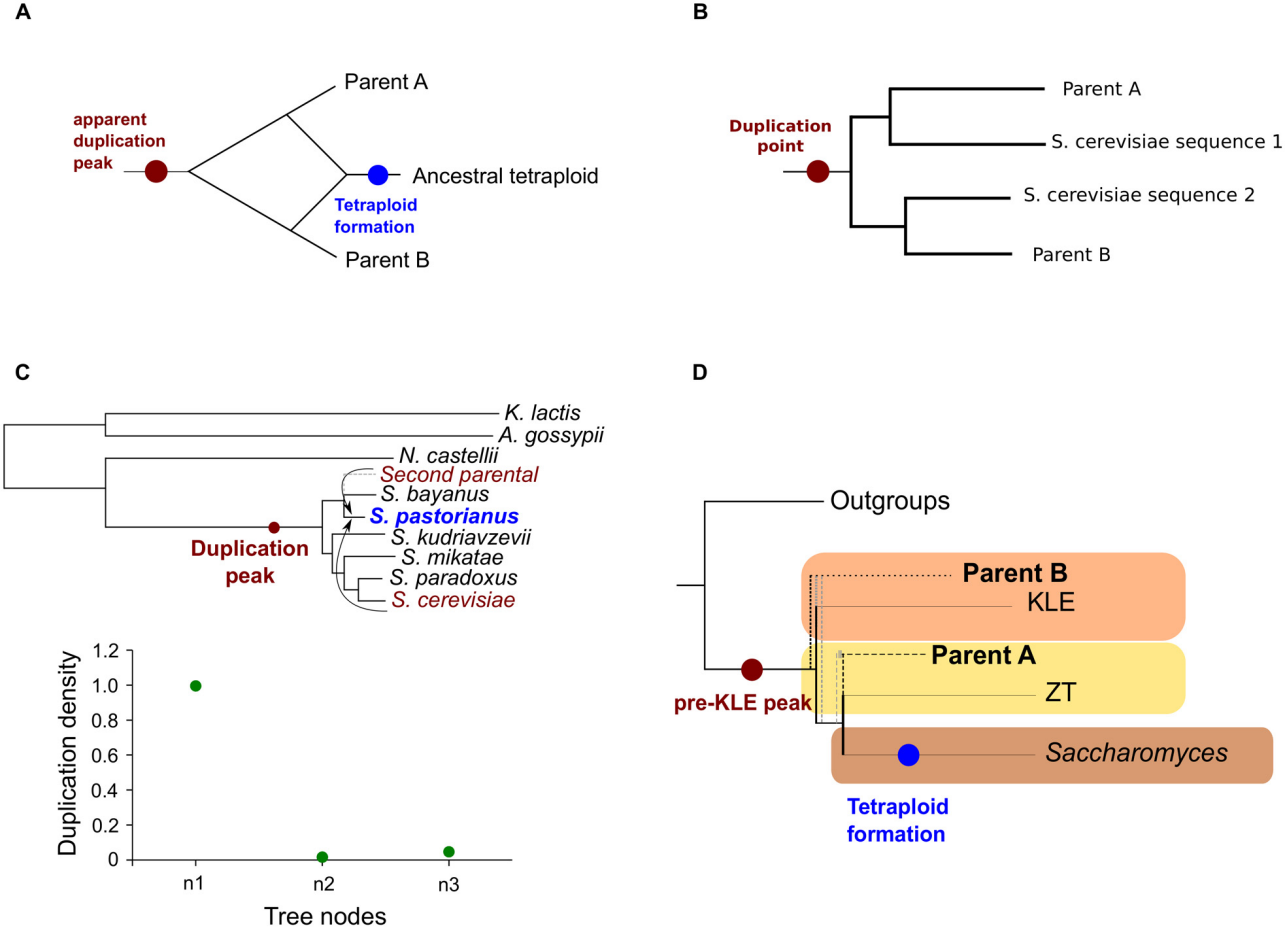
Assessment of hybridization parental lineages. (A) Schematic example of how the pre-KLE node is found in the common ancestor of the two parents, whereas the tetraploid was formed afterwards. (B) Schematic example of duplication inference at the pre-KLE position from a gene tree with genes coming from two parentals. (C) Top: maximum likelihood species tree representing the evolution of *S*. *pastorianus*. The tree was obtained using the same approach as the tree in Fig 1: 215 alignments from genes present in single copy in *S*. *pastorianus* and with orthologs in all the species considered were concatenated and analysed using maximum likelihood. Bootstrap support was maximal (100%) in all branches. The red dot represents the branch where the duplication peak can be found. Bottom: Graph representing the duplication density (duplications per gene per branch) found at three different branches in the species tree. (D) Schematic representation of the inferred positions of the putative parents, related to the main fungal groups considered. The most likely position of the two parents is marked in a black, dashed line, while a second possible position is marked in a grey, dashed line. Data on which this figure is based are provided in S1 Data.

We further considered possible evolutionary scenarios that could result in the observed patterns of ancestral duplications seen for the ohnologs. We reasoned that an interspecies hybridization would result in phylogenetic patterns reminiscent of duplications that would be mapped to the common ancestor of the two hybridizing species (Fig 2A), providing a possible scenario to explain our puzzling results. The process of hybridization originates a new lineage by bringing together two diverged genomes. Orthologous genes coming from each of the parental species would appear as paralogs in standard analyses, since they are homologous genes encoded in the same genome [35]. A phylogenetic analysis, however, would map the apparent duplication to the time of divergence of the two parental species (Fig 2B). This necessarily predates the time of the formation of the hybrid: that is, the hybridization point does not coincide with the point at which the apparent duplications would be mapped. As we will see below, our hypothesis is that the hybridization may have shortly predated the actual WGD point (i.e., occurred at node n5 in Fig 1).

In support of this, we calculated the duplication densities on the well-studied yeast interspecies hybrid *S*. *pastorianus* [36]. This species is the result of a recent hybridization between *S*. *cerevisiae* and *S*. *eubayanus* [36]. The sequenced genome of *S*. *bayanus* is the closest related genome to *S*. *eubayanus*, and therefore we expect the highest duplication peak to appear at the common ancestor between *S*. *cerevisiae* and *S*. *bayanus*. The duplication density analysis, as predicted, yielded an apparent duplication peak at the common ancestor *S*. *cerevisiae* and *S*. *bayanus*, but not at the lineage where the hybridization and the doubling of the genome is known to have occurred (Fig 2C).

**Fig 3:**
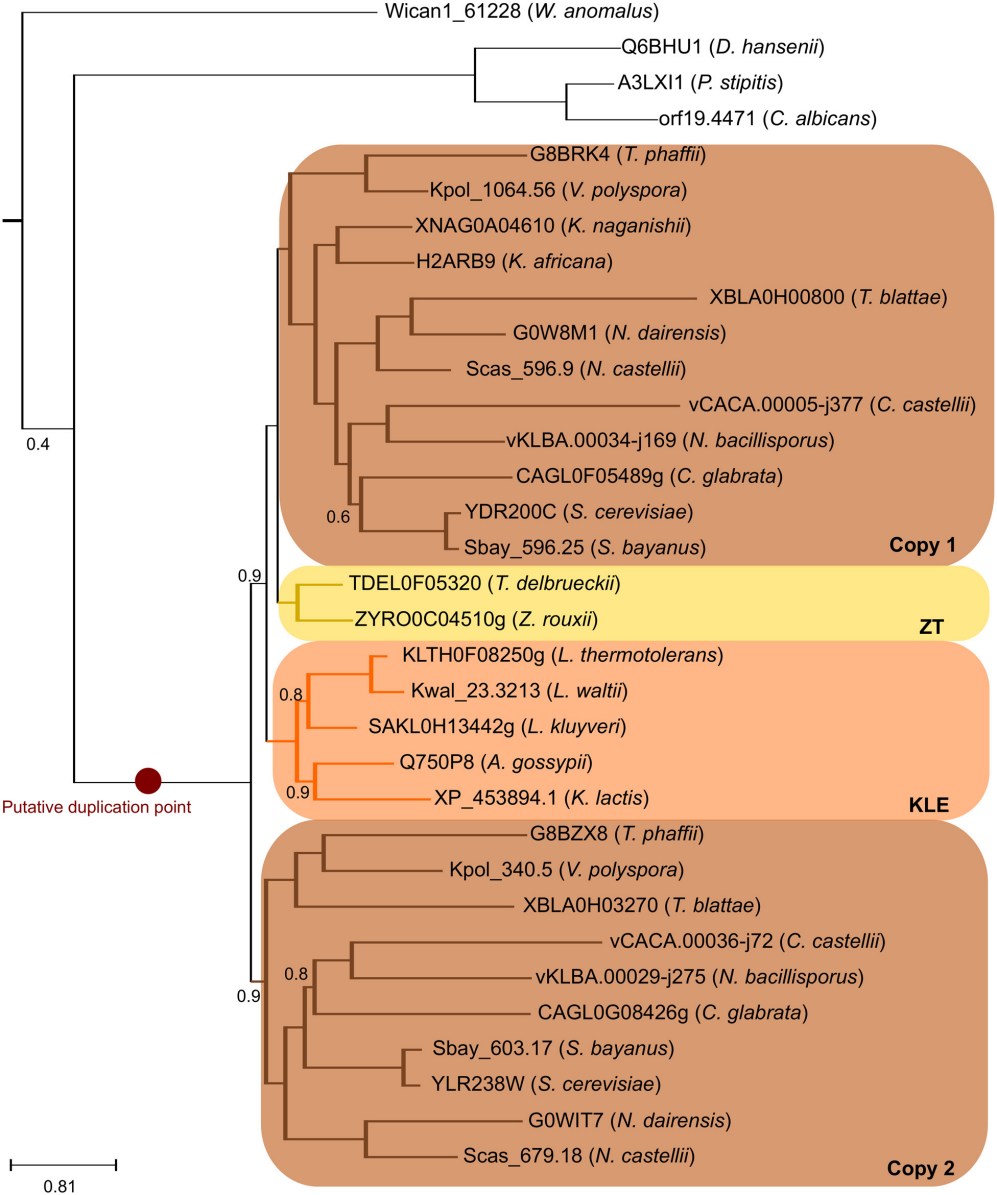
Example of a phylogenetic tree with a topology that supports the hybridization scenario. Example of a phylogenetic tree in which two copies were retained after the formation of the tetraploid. One copy shows topology A, while the second copy shows topology C. The putative duplication event is indicated in red. Support for the topology is indicated as aLRT values.

**Fig 4:**
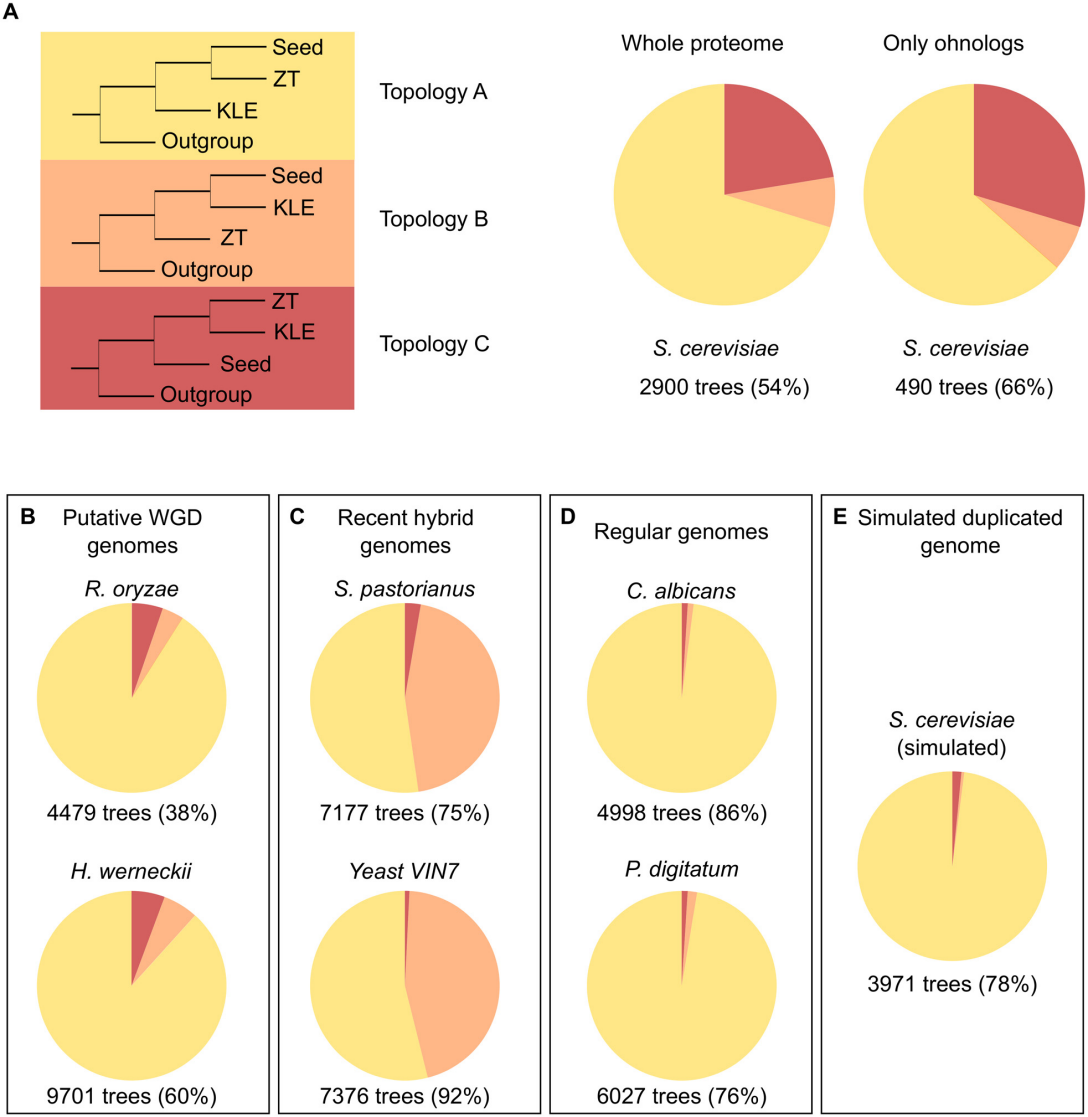
Topological analysis of polyploids. (A) Phylogenetic representation of the three possible topologies regarding the placement of the post-WGD and the two parental sequences (ZT and KLE). The pie chart on the left represents the average percentage of trees found in all the *S*. *cerevisiae* reduced phylomes that supported each topology. The average was calculated from the results of the different reduced phylomes (see S9 Fig). The pie chart on the right represents the same pie chart but only using those trees within the reduced phylomes that contain *S*. *cerevisiae* proteins that have a conserved ohnolog. The average was calculated from the results of the different reduced phylomes considering only trees in which the *S*. *cerevisiae* sequence has a conserved ohnolog (see S10 Fig). Numbers below the pie charts indicate the average number of trees that passed the filters and the percentage it represents when compared to the total. (B) Same pie charts as in A but for two genomes that underwent a WGD. (C) Same pie charts as in A but for two genomes that underwent a hybridization. (D) Same pie charts as in A but for two genomes that have not been duplicated. (E) Same pie chart as in A but for the simulated phylome. Data on which this figure is based are provided in S1 Data.

The results found for the *S*. *cerevisiae* lineage could thus be readily explained by a past hybridization between lineages diverging just after the observed peak and before the post-WGD species. Considering this and the current genomic sampling, species close to, but not necessarily within the KLE and ZT clades, would be the prime suspects of potential partners in the proposed ancestral hybridization (Fig 2D). To explore this possibility further, we inferred properties of the two putative parental lineages from the current genomic sampling. We did so by inspecting individual *S*. *cerevisiae* gene phylogenies in the above-mentioned phylomes (see Fig 3 as an example) and by measuring phylogenetic affiliations using phylomes reconstructed with reduced taxonomic sets (see S2 and S3 Tables). Phylogenetic affiliations were measured by scanning the gene tree topologies to examine the species contained in the sister groups (i.e., neighbouring clades) of the sequences from post-WGD species (see Materials and Methods). We categorized them according to one of the two lineages that diverged after the pre-KLE peak and the origin of the post-WGD species: the KLE clade and the ZT clade. From now on, we consider the ZT cluster as the extant clade closest to one of the parents (parent A), while the KLE cluster will be considered as the closest to the other parent (parent B). Although, for simplicity, we refer to ZT and KLE clades as parental lineages, it must be clearly stated that it is our understanding that the actual parents may have been close to, but not necessarily within, these clades. Accordingly, three possible topologies can be considered: two in which the *S*. *cerevisiae* seed sequence groups with either parental species (A or B, respectively) and a third one in which the *S*. *cerevisiae* sequence has the two parental lineages as a sister group (C) (see Fig 4A). Our results (Fig 4A) indicated that a large majority (60%–82%, depending on the choice of species used in the reduced phylome; see S9 Fig) of the trees showed a topology congruent with the currently accepted phylogeny, i.e., the post-WGD species grouping with the ZT clade. When only the trees that contain *S*. *cerevisiae* proteins with a conserved ohnolog are considered, the results remain very similar (see Fig 4A) (54%–78%, depending on the choice of species used in the reduced phylome; see S10 Fig). This suggests that this or a related lineage would have been involved in the hybridization (parent A) and that genes derived from this parental species constitute a majority of the genome in extant post-WGD species. In contrast, a remarkably low fraction of genes showed an affiliation only to the KLE lineage (4%–14%), whereas a larger percentage (14%–28%) of genes had as a sister group a combination of the two putative parental clades (C). This would suggest that one of the actual parental lineages did not belong to the KLE but rather diverged before. The analysis repeated using different phylogenetic methodologies confirmed these results (S11 Fig).

The high percentage of trees supporting the A topology could be the result of total or partial gene conversion, which is common in recent hybrids [37]. We can only clarify this matter by analysing gene trees that contain pairs of conserved ohnologs. Depending on the distribution of the two ohnologous genes when compared to KLE and ZT, we can distinguish between nine different topologies (see S12 Fig). Forty percent of the trees contained a topology in which the two yeast ohnologs grouped together (topologies A–A 1, B–B 1, and C–C 1). This could be due to total or partial gene conversion from one of the parents to the other. The gene conversion events seem to favour genes from parent A, since in 30% of the mentioned cases both retained genes are more closely related to this parent. We performed sequence evolution simulations including different degrees of gene conversion to estimate what levels would be necessary to alter the tree topology (see Materials and Methods). In our settings (S13 Fig), conversion of 25% of the gene sequence was sufficient to lead to a higher probability of the duplication being mapped to a younger node. Thus, gene conversion, which renders duplications to appear younger, has a much larger effect than LBA.

These analyses underscore the difficulty of correctly determining the position of parent B. There is a strong signal for the parent B to have diverged just before the KLE clade (shown by topology A–A 2 and B–B 2), which is present in 33% of the trees. As we will discuss below, we consider that recombination between the two parental subgenomes, including total or partial gene conversion, must have been common in the period following the hybridization, explaining not only the bias in descent among ohnologs and singletons but also the widespread mixture of phylogenetic signals in gene trees that is typical for this clade [20].

The availability of genomes from fungal species in which recent hybridizations or WGDs have been described allows us to assess the patterns of phylogenetic affiliations and compare them with the patterns observed for *S*. *cerevisiae*. On the one hand, *Rhizopus delemar* [38] and *Hortaea werneckii* [39] are thought to have undergone a recent WGD. On the other hand, *S*. *pastorianu*s [36] and the wine strain *S*. *cerevisiae* x *S*. *kudriavzevii* VIN7 [40] are recognized as recent hybrids for which the putative parental species are known. It is important to remark that some of the described WGD species may indeed be as well the result of hybridizations, as it is proposed here for the post-WGD clade, but that the current sampling of species prevents the detection of the alternative parental signals. We reconstructed the phylomes of these four species (see S2 Table) and computed phylogenetic affiliations as explained above, but adjusting A and B to the known parents or the corresponding neighbouring clades. Putative WGD species showed a clear dominance of the immediate preceding clade (Fig 4B). The recent hybrids, on the other hand, presented a split topology distribution, with roughly half of the trees supporting the A topology and another half supporting the B topology (Fig 4C). This clearly provides evidence of the dual origin of these species. As negative controls, we examined the phylomes of two species without anomalous ploidy, *Candida albicans* and *Penicillium digitatum* (Fig 4D) [41], and the above-mentioned simulated yeast phylome in which one of the ohnologs was evolving at a faster rate (Fig 4E). This analysis shows that hybrids present a clear dual pattern of phylogenetic affiliations when the gene phylogenies are examined in the presence of the two parental lineages. This pattern is clearly distinct from what is in genomes with normal ploidy or in recent WGDs. This dual pattern is also present in the analysis of the yeast genome. Of note, in this case the two alternative phylogenetic affiliations are not equally represented. This difference with respect to recent hybrids can be attributable to the larger period of time since the hybridization and the preferential loss or conversion of genes coming from one of the parental lineages, which necessarily altered the balance between the two phylogenetic affiliations.

### Reinterpreting Gene Order Conservation in Light of Hybridization

As mentioned above, inferred ancestral collinearity has been used to favour simpler WGD scenarios involving autopolyploidization [11]. However, such studies indistinctly used KLE and ZT clades to infer ancestral gene arrangements and thus could not inform about differences between the putative parents. Although the position of the parental species cannot be ascertained with confidence, we can take KLE and ZT clades as the two extremes of their possible divergence. We therefore assessed the level of micro- and macrosynteny conservation among the KLE, ZT, and post-WGD clades, by considering them separately. To do this, we reanalysed the information of orthology and syntenic blocks provided by YGOB [42]. We first assessed the differences between ZT and KLE by searching for gene arrangements conserved within ZT and KLE, but different between the two groups. These differences can be considered ancestral to the two groups and thus likely present at the time of the proposed hybridization. Only 32 cases of broken synteny and 11 translocations of a single gene were noted (S4 Table). When searching for these synteny breaks in post-WGD species, we found that they had inherited the arrangement present in either KLE or ZT in similar amounts (15 and 17, respectively) (S5 Table). Of note, the patterns shared by KLE and post-WGD species could result from lineage-specific rearrangements in the lineage leading ZT clade so we cannot unequivocally impute them to the hybridization. This result is consistent with the absence of disagreements between syntenic ohnologous blocks noted earlier [11].

**Fig 5:**
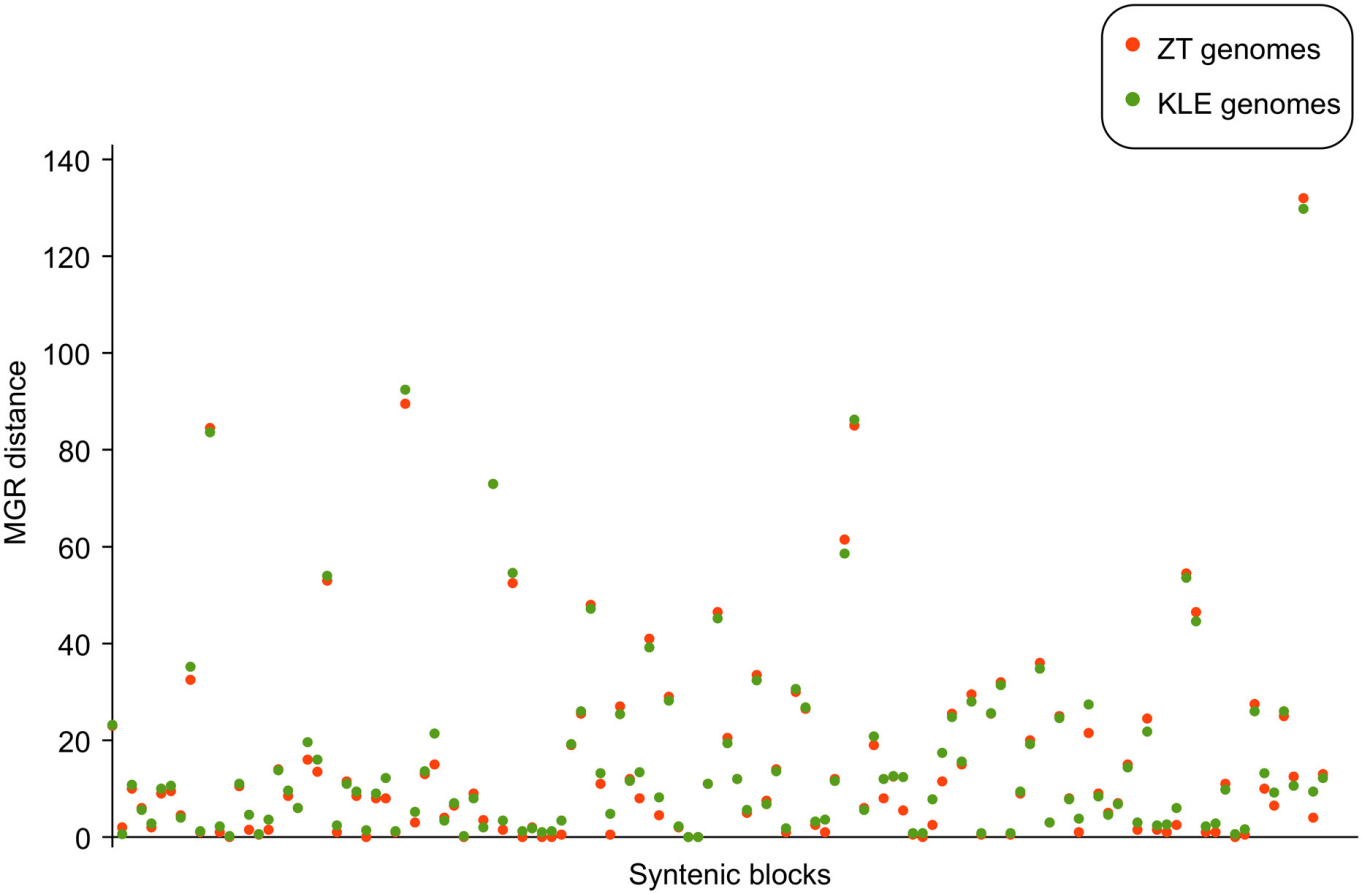
Genome rearrangements of syntenic blocks. Average number of genome rearrangements as calculated by MGR (Multiple Genome Rearrangements) [43] for each syntenic block inferred from Gordon et al. [11]. Orange dots represent the number of rearrangements between the *S*. *cerevisiae* block and the orthologs found in the ZT genomes, while the green dots show the same value for the comparison between the *S*. *cerevisiae* genome and the KLE genomes. Data on which this figure is based are provided in S1 Data.

However, an autopolyploidization scenario would predict a larger number of shared syntenic arrangements between the post-WGD and its closest clade (ZT). Furthermore, the absence of disagreements in such a small number of blocks can be explained by other factors, including gene conversion, so it cannot be considered a definitive proof of autopolyploidization. In addition, we found that the number of conserved pairs of adjacent orthologs between KLE and ZT clade was high, as was the number of conserved pairs between post-WGD species and any of the KLE and ZT clades (S6 and S7 Tables). Finally, we found no differences in terms of the minimal amount of rearrangements [43] between each *S*. *cerevisiae* syntenic block [11] and those in either ZT or KLE species (Fig 5). These results speak for the high collinearity of the two putative parental clades at the proposed time of hybridization (see S4 and S5 Tables), which is congruent with the small divergence time estimated between the two clades at the time when the post-WGD clade originated (S14 Fig). In addition, considering the high level of collinearity between the ZT and KLE clades, and the lack of differences in terms of synteny conservation when compared to *S*. *cerevisiae*, the proposed hybridization is as compatible with the observed level of conserved synteny between duplicated blocks as a simpler autopolyploidization scenario. Recent yeast hybrids have been shown to present extensive recombination between parental genomes, including total or partial gene conversion [37,44,45], which breaks the initial correlation between phylogenetic origins of neighbouring genes and removes sequence and structural differences between homologous chromosomes. This and extensive differential gene loss and genome rearrangements that have occurred within the post-WGD clade have presumably eroded the few initial differences between the two parents that we can reconstruct. We conclude that, given the similar levels of collinearity implied by both scenarios and the confounding effects of extensive gene loss, homologous recombination, gene conversion, and genome rearrangements, synteny cannot be used in this case to disentangle whether the WGD was triggered by an autopolyploidization or a hybridization event.

### A Proposed Model for the Origin of the Yeast WGD through Hybridization

The proposed hybridization is a very ancient event, and thus, the remaining signal must be necessarily weak. We have shown that gene order differences between the putative parental species involved in the hybridization were extremely low, and we consider that this signal may have been completely eroded, which explains why the hybridization was not evident from earlier analyses based on synteny. Our phylogenetic results, however, do provide clear support for the existence of an ancient interspecies hybridization and are not compatible with a simple autopolyploidization scenario. The observed phylogenetic affiliations in ohnologs and singletons, biased towards one of the putative parental lineages, as well as the absence of synteny disagreements in ohnologous blocks, can be reconciled with the assumption that the proposed hybridization was followed by widespread recombination events between the two parents subgenomes, some of which would have led to partial or total gene conversion. As noted before, this process is common in recent yeast hybrids [37,44,45], and it is natural to expect that this would have occurred in an ancient hybridization. Notably, hybridization followed by recombination between parental subgenomes also explains another long-held observation of the post-WGD clade: that there is a variable mixture of disparate phylogenetic signals present across different gene trees [20,22].

**Fig 6:**
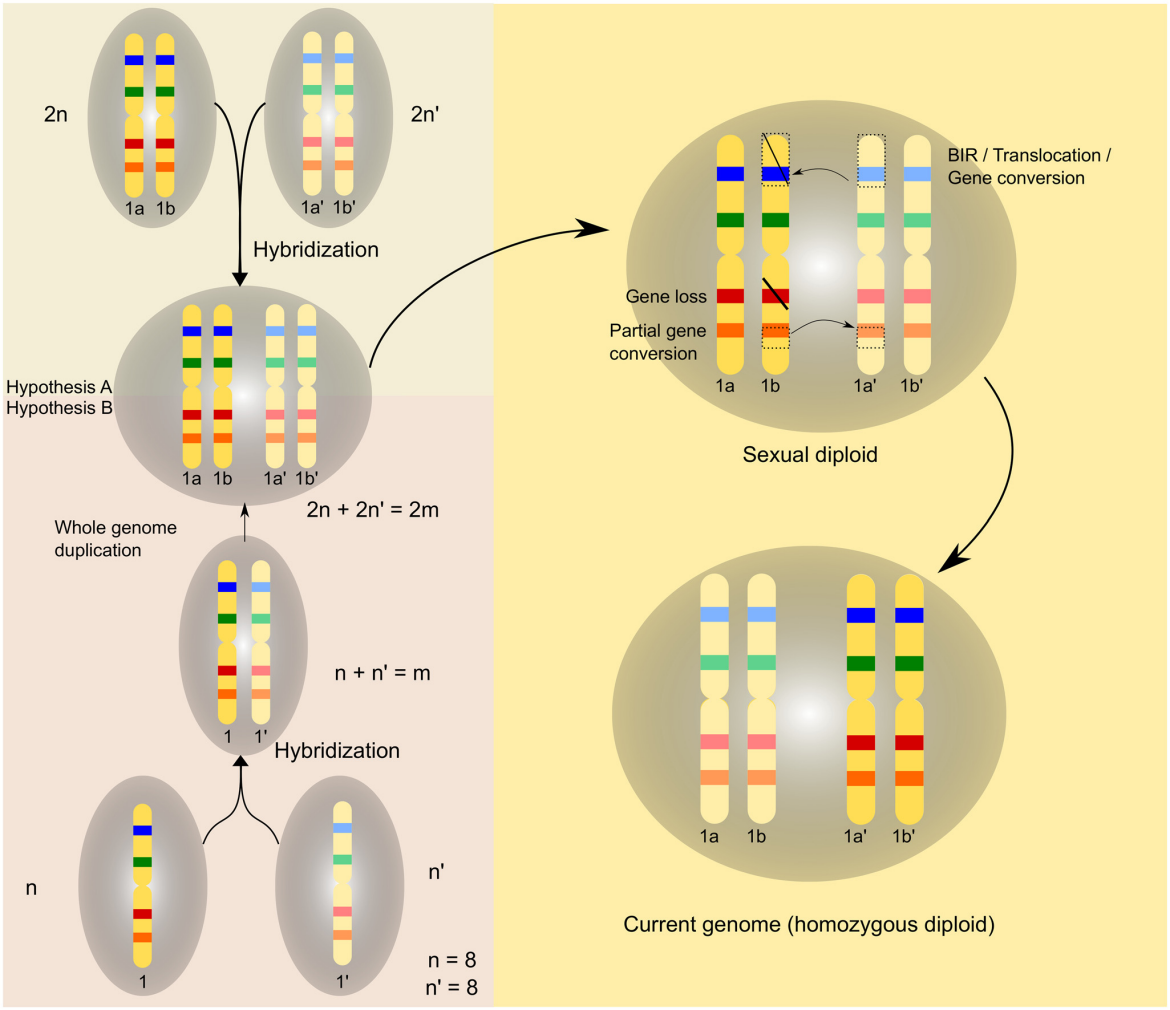
Hybridization scenarios: Schematic representation of possible scenarios of WGD following interspecies hybridization. Homeologous chromosomes for the two hybridizing species are coloured in yellow. Bands on the chromosomes represent genes. Pairs of genes of the same colour are paralogs. Hypothesis A shows the fusion of two diploids and the formation of the allotetraploid. Hypothesis B shows the mating of two haploids and the posterior WGD that leads to the formation of the allotetraploid. The upper right cell contains two pairs of meiotic homologous chromosomes (1a–1b and 1aʹ–1bʹ) and shows the different events that have affected the hybrid during diploidization. The cell at the bottom right represents the current yeast genome as a homozygous diploid.

Our results also indicate that an apparently more ancestral duplication peak occurred in addition to duplications around the expected WGD point. We hypothesize that the occurrence of these two rare events in the same lineage is not the result of coincidence. We propose two possible scenarios that naturally link the two events and explain the observed patterns (Fig 6). In the simplest scenario, two diploid cells from distinct species form an allotetraploid. Subsequent recombination and massive gene loss would render a lineage in which the number of chromosomes has effectively doubled. In this case, hybridization directly results in the observed WGD, because a fraction of the final gene set is retained as “ohnologous” pairs, either from the same or from different parental species. Alternatively, two haploid cells from different species form an allodiploid. Such hybrids are largely unstable and cannot undergo the sexual cycle, but they can propagate clonally [46]. An additional duplication by autopolyploidization would stabilize the hybrid by enabling meiotic recombination. This mechanism, which also prevents backcross with the parental lineages, has been proposed as a necessary step to stabilize some interspecies hybrids [47] and is a scenario commonly considered in recent plant hybrids [48]. Both scenarios cannot be distinguished with the data at hand, but the ability of haploid cells to fuse through mating provides a possible mechanism for the latter. Further investigation of how these two mechanisms participate in the formation of natural hybrids is necessary [46]. Importantly, some of the steps proposed by our model were also contemplated in models considering autopolyploidization scenarios [10]

### Concluding Remarks

Our results provide compelling evidence for an ancient hybridization in the yeast lineage and bring about novel implications in our understanding of the evolution of eukaryotic genomes and the origin of functional divergence after WGDs. Remarkably, besides the pattern of ancient duplications, the proposed model provides plausible explanations to other common observations in the post-WGD clade. The phylogenetic relationships within and around the post-WGD clade have always been difficult to resolve, and a great diversity of phylogenetic histories among different genes has been noted [20,22]. A chimeric origin of the clade, combined with events of recombination between genes from different parents—as observed in current hybrids [44,49]—would readily explain an increased variability in phylogenetic signals recovered from different genes. Such intragenic recombinations, together with full gene conversion and differential gene loss, may as well partially explain the observed dispersion of the phylogenetic mapping of duplications from syntenic ohnologs around the expected WGD point. Furthermore, ohnologs have been shown to present selection pressures intermediate of singleton genes and those from small-scale duplications of a similar age [50]. Finally, notable exceptions to expectations from the gene balance hypothesis, which posits that WGD would favour duplications of entire complexes rather than single subunits, have been noted [51]. Most of these observations have been interpreted in the light of an assumed rapid sequence and functional divergence after duplication. However, under a hybridization scenario, a fraction of the predicted ohnologs originate from distinct species, and thus, sequence and functional differences are expected from the start. In contrast, an autopolyploidization scenario poses the problem of how reproductive isolation was achieved and faces the lack of a clear selective advantage before neo- or subfunctionalization occurs. Interspecies hybridization brings together different physiological properties and isolates sexually the newly formed lineage, hence providing an initial selective advantage to explain observed WGDs in eukaryotes. Considering the widespread presence of hybrids among current species, this scenario should also be considered when interpreting ancient polyploidies. The proposed approach and an increased genome sampling around the relevant lineages will enable testing the possible implication of interspecies hybridization in other eukaryotic WGDs.

## Materials and Methods

### Sequence Data

Proteomes were downloaded from their original databases (S8 and S9 Tables). The proteomes of *S*. *pastorianus* and *H*. *werneckii* were not available. We thus downloaded the genomes and predicted their proteomes using Augustus [52]. The final *S*. *pastorianus* [36] and *H*. *werneckii* [39] proteomes comprised 11,460 and 20,509 proteins, respectively.

### Phylome Reconstruction

Phylomes—complete collections of phylogenetic trees for each gene encoded in a given genome—were reconstructed using the automatic pipeline described in Huerta-Cepas et al. [23]. Briefly, the pipeline starts with a seed genome and proceeds as follows: for each protein encoded in the seed genome, a Smith-Waterman similarity search was performed against a database containing the proteomes listed above. Results were then filtered based on e-value (<1e-05) and sequence overlap (>50% coverage over the query sequence). The query and the selected hits (homologous sequences) were then aligned using a sophisticated multiple sequence alignment strategy in which three different alignment programs were used (Muscle v3.8 [53], Mafft v6.712b [54], and Kalign v2.04 [55]) to align the sequences in forward and reverse orientation. The resulting six alignments were combined into a consensus alignment using M-coffee [56]. This alignment was then trimmed to remove poorly aligned columns with trimAl v1.3 [57] using a consistency-score cutoff of 0.1667 and a gap-score cutoff of 0.9. Trees were reconstructed using the best-fitting evolutionary model. The selection of the model best fitting each alignment was performed as follows: a neighbour joining (NJ) tree was reconstructed as implemented in BioNJ [58]; the likelihood of this topology was computed, allowing branch-length optimization, using seven different models (JTT, LG, WAG, Blosum62, MtREV, VT, and Dayhoff), as implemented in PhyML v3.0 [28]; the two models best fitting the data, as determined by the AIC criterion [59], were used to derive maximum likelihood (ML) trees. Four rate categories were used, and invariant positions were inferred from the data. Branch supports were computed using an aLRT based on a chi-square distribution, as implemented in PhyML [60].

S2 Table lists the complete phylomes reconstructed for this project. Seven complete phylomes were reconstructed using *S*. *cerevisiae*, *C*. *glabrata*, *V*. *polyspora*, *S*. *pastorianus*, *H*. *werneckii*, the yeast *S*. *cerevisiae* VIN7, and *R*. *delemar* as seed species. These phylomes have been deposited in phylomeDB (http://phylomedb.org [61]). A simulated phylome using *S*. *cerevisiae* as seed was also reconstructed (see below). In addition, a total of 18 reduced phylomes were reconstructed (see S3 Table; http://genome.crg.es/~mmarcet/yeast_hybrids/phylome_table.htm). In these reduced phylomes, for the seed species, only one sequence was present in the tree; all paralogs for this species were removed to ensure that a clear phylogenetic position could be established. Finally, two previously reconstructed phylomes, stored in phylomeDB, were used for comparative purposes: *C*. *albicans* (phylomeID: 205) and *P*. *digitatum* (phylomeID: 150) [41]. Phylomes were scanned using ETE v2.2 [62], which implements all the algorithms described here.

### Species Tree Reconstruction

The reference species tree shown in Fig 1 was reconstructed using a multigene concatenation method. From the *S*. *cerevisiae* phylome, we selected 516 protein-coding genes found in single copy across the 26 species considered. Their protein alignments were then concatenated, resulting in a combined alignment of 285,507 positions. An ML phylogenetic tree was then reconstructed using PhyML v3.0 [28] using the LG model. Four rate categories were used, and invariant positions were inferred from the data. Bootstrap support was calculated based on 100 replicas. All nodes were fully supported (100% bootstrap). The species tree presented in S3 Fig was reconstructed using the same data as the previous tree, but enforcing the desired topology when reconstructing the tree. For the *S*. *pastorianus* tree (Fig 2C), 215 genes were selected from the phylome, and the final alignment contained 117,408 amino acids. The same methodology was used to reconstruct the tree.

### Calculation of Duplication Density per Branch

Each tree in a phylome was scanned to detect and date duplications using a phylogeny-based algorithm described earlier [16]. In brief, this algorithm traverses the tree and uses a so-called species-overlap algorithm to detect duplication nodes. Duplication nodes are defined as those nodes where the two daughter branches share at least one species. The relative age of this duplication is assumed to be at the last common ancestor of the species diverged after the duplication (i.e., those contained in the two daughter branches). Each duplication was then mapped onto the corresponding ancestral lineage in the species tree. The total number of duplications was divided by the total number of trees that were rooted at a deeper branch in the species tree (i.e., those that are informative for the evaluated lineage). For instance, to estimate the duplication density at the WGD branch, only trees that contain at least one pre-WGD species were considered. S1 Fig shows a schematic representation of the duplication mapping process. This analysis was performed using three different phylomes, in which *S*. *cerevisiae*, *V*. *polyspora*, and *C*. *glabrata* were used as seed, respectively. For each phylome, two different datasets were used. In the first one, all the trees in the phylome were used (see Fig 1B, green dot, and S2 Fig, lighter dots), the second was based on trees in which a pair of retained ohnologs was present, and only the duplication node leading to the two seed ohnologs was used (see Fig 1B, yellow dot, and S2 Fig, darker dots). Ohnologs were obtained from YGOB [42], which uses a synteny criterion combined with sequence similarity but is not phylogenetically informed. Only trees that contained both ohnologs were considered. This second set ensured that the duplication density was not affected by duplications not related to the WGD event. We plotted the correlation between duplication densities and branch lengths.

### Comparison of Sequence Divergence

We mapped the duplication event of the two ohnologs to the species tree and only kept those *S*. *cerevisiae* sequences whose duplication point mapped to the WGD node or to the pre-KLE node (see Fig 1). Only trees that contained at least one ZT sequence, one KLE sequence, and one out-group sequence were considered. Blast scores were normalized by dividing the blast score obtained when searching from a seed yeast protein to the ohnolog pair by the blast score obtained from searching the seed yeast protein to itself. In a separate analysis, pairwise alignments of the conserved ohnologs were reconstructed using Muscle v3.8 [53]. The Kimura distance between the two sequences was calculated using protdist as implemented in the phylip package [63]. The frequency of distances of the two different distributions and blast score frequencies were plotted with R [64]. Significance of the difference in distributions was assessed using a two-sample Kolmogorov-Smirnov test (see Fig 1C). The two populations were significantly different, with a *p*-value for the blast scores of 2e-04 and for the Kimura distance of 2.9e-05.

### Estimation of Divergence Times

PL-R8s [14] was used to assess the divergence times in the concatenated species tree (S14 Fig). Smoothing parameter was estimated using cross validation. The divergence between *S*. *cerevisiae* and *C*. *albicans* (235 MyA as estimated by Douzery et al. [65]) was used as calibration point. The same protocol was used in individual trees that contained two ohnologous pairs. Trees were pruned so that they only contained the closest sequence belonging to each ZT-KLE group. The frequencies of ages (see Fig 1C) were plotted using R [64]. The two populations were significantly different, with a *p*-value of 4.5e-07.

### Tree Reconciliation

Notung v2.6 [27] was used to reconcile the same set of trees used above to the species tree obtained from the concatenation of 516 proteins (see above). Once the two trees were reconciled, we used the option to estimate upper and lower bounds to obtain the time when the duplication of the two *S*. *cerevisiae* ohnologs had taken place. Only estimates that had a definite upper and lower bound that could be mapped to a single branch of the species tree were considered. The number of trees that mapped the duplication onto a given branch was divided by the total number of trees in order to obtain the duplication density.

### Reconstruction of Trees Using Different Phylogenetic Methods

A set of 846 trees were selected from the *S*. *cerevisiae* phylome where pairs of conserved ohnologs were found, as predicted by YGOB [42]. The alignments were taken from the phylome reconstruction done previously. Then, for each tree, several additional phylogenetic reconstruction methods were used. Fasttree [30] was used with default values. PhyML [28] was run again three times; in all cases, four rate categories were applied and invariant positions were calculated from the data. The first time the CAT model C20 was used [32], the second time the Covarion model [33] was used (—cov_free –cov_ncats = 3), and finally, the same models as in the phylome were used, but instead of using NNI to estimate the tree topologies, SPR was used. For the three methods, the aLRT support was calculated. A fourth run with PhyML was performed using the same method as during the phylome reconstruction, but instead of calculating aLRT support values, bootstrap values based on 100 replicates were computed. RAxML [29] was applied using the PROTGAMMALG model and rapid bootstrapping to obtain the branch support. The SH support as implemented in RAxML was calculated over the same set of trees. A Bayesian approach was also used. Phylobayes [31] was used to reconstruct the trees; for each tree, two chains were run for a minimum of 500 cycles; every 100 cycles, the two chains were automatically compared; and if the discrepancies were lower or equal to 0.3 and the effective sizes were larger than 50, the process was stopped. The majority rule consensus, annotated with posterior probabilities, was obtained for each tree.

For each set of trees, the duplication density for the duplication point that led to the diversification of the two *S*. *cerevisiae* ohnologs was calculated. Results can be found in S7 Fig. Only nodes in which the support value at the common ancestor of the two ohnologous sequences has an aLRT > 0.95 or a bootstrap > 95 or a posterior probability > 95 were considered.

### Tree Reconstruction without Out-groups

The same set of 846 trees was reconstructed with no out-group sequences using the same methodology used for phylome reconstruction (see above). The trees included only the post-WGD sequences and the ZT and KLE sequences. Trees were then checked to see whether the two *S*. *cerevisiae* ohnologs had a common ancestor that contained no sequences of the ZT and KLE groups, therefore giving support to the WGD, or if they had sequences of either group in between. Only trees in which the common ancestor of the two *S*. *cerevisiae* sequences has a support over 0.5 were considered. The same procedure was performed in the same set of trees taken from the phylome. Out-groups in this case were used to root the tree, and then the same analysis was performed. Fifteen percent of the trees gave a different prediction when the two methodologies were performed.

### Simulations to Test for Long Branch Attraction

For each sequence encoded in the yeast genome that had one-to-one orthologs in all the species considered, alignments obtained during the phylome reconstruction were trimmed to remove all positions with gaps. The number of species considered was reduced to 12, including *S*. *cerevisiae*, all the species belonging to the ZT and KLE clades (*T*. *delbrueckii*, *Z*. *rouxii*, *Kluyveromyces lactis*, *A*. *gossypii*, *Lachancea kluyveri*, *L*. *thermotolerans*, and *L*. *waltii*) and four outgroups (*Schizosaccharomyces pombe*, *Yarrowia lipolytica*, *C*. *albicans*, and *Wickerhamomyces anomalus*). The species tree (see Fig 1) was pruned to match this set of species. The existing tree branch that contained *S*. *cerevisiae* was bifurcated to create two new branches containing simulated yeast paralogs. The first branch contained the original *S*. *cerevisiae* leaf, but its branch length was cut in half. The second branch contained a new *S*. *cerevisiae* leaf with a branch length ten times longer than the original. Each protein was then made to evolve along this tree using Rose [66]. Tree-puzzle [67] was used to obtain the mutation frequency observed at each site of the alignment. Tree-puzzle was run with the JTT model; the gamma distribution was estimated from the data using 16 rate categories. These mutation frequencies produced very conserved, unrealistic alignments with few mutations; therefore, the frequencies were multiplied by 20, resulting in more realistic alignments. Indel frequency was set at 0.0003. The resulting sequences were then treated as a newly simulated phylome, which was run through the phylome pipeline. Duplication densities were then mapped onto the species tree (see S8 Fig).

### Topology Scanning: Reduced Phylomes

Reduced phylomes were reconstructed in such a way that they contained only one species for the post-WGD (seed species), one species for the ZT clade, and one for the KLE clade, in addition to three outgroups (*C*. *albicans*, *Y*. *lipolytica*, and *S*. *pombe*). In addition, for the seed species, only the seed sequence was included; other paralogs in this organism were excluded from the tree. A reduced phylome was reconstructed for each pair of ZT-KLE species. Three post-WGD species were used as seed (*S*. *cerevisiae*, *C*. *glabrata*, and *V*. *polyspora*) (see S3 Table). For each seed sequence in the reduced phylomes, the sister branch was analysed. First, trees were excluded if they did not have any homologs in ZT, in KLE, or in any of the out-group species. Then, the support of the clade containing the seed sequence and its most immediate neighbouring clade was evaluated using aLRT values. Only clades with support higher than 0.95 were considered. The phylogenetic affiliation of the seed sequence was classified into one of the following groups, according to the species that were present in its neighbouring clade (i.e., sister branch): A, the species located in the sister branch belonged to the ZT clade formed by *Z*. *rouxii* and *T*. *delbrueckii* (putative parent A); B, they belonged to the clade formed by *A*. *gossypii*, *K*. *lactis*, *L*. *thermotolerans*, *L*. *waltii*, and *S*. *kluyveri* (KLE clade, putative parent B); and C, they contained a mix of both clades. This was done for the whole phylome (S9 Fig) and for the trees in which the seed sequence was part of a conserved ohnologous pair (S10 Fig). Analysis was repeated across several phylogenetic methods (see above) (S11 Fig).

Topologies of pairs of ohnologs were assessed by reconstructing the trees including the ohnologous pair to those trees that already contained a sequence with a conserved ohnolog. Depending on the relation between the two ohnologs and the chosen KLE and ZT parent sequences, we distinguish between nine possible topologies: A–A 1, A–A 2, B–B, B–B 2, C–C, C–C 2, A–B, A–C, and B–C (see S12 Fig).

### Topology Scanning: Complete Phylomes

For the complete phylomes used (*C*. *albicans* phylome, *H*. *werneckii* phylome, *S*. *pastorianus* phylome, *R*. *delemar* phylome, and *S*. *cerevisiae* x *S*. *kudriavzevii* VIN7 phylome), the two groups of species situated closest to the seed species according to the species tree were used as parental species unless the parental species were known (see S2 Table). Trees were then pruned so that only the seed, the two parents, and out-groups were kept. ETE v2.2. [62] was then used to analyse the sister branch (i.e., neighbouring clade) to the seed sequenced. Sequences were classified as explained above.

### Simulations in the Presence of Gene Conversion

For the same set of sequences used in the LBA simulation (see above), we used ROSE [66] to make the sequences evolve along a species tree that contained two *S*. *cerevisiae* sequences. The branch lengths of the tree were inferred by selecting those genes that had an A topology and a C topology and were consistent across different phylogenetic methods. Two species trees were derived from these two sets of genes, and branch lengths were mapped onto our simulated species tree. Once sequences were reconstructed, sets of genes affected by different levels of gene conversion were reconstructed. For each percentage of gene conversion, one yeast sequence was taken for each set of sequences, and a given percentage of its sequence was replaced by the same fragment of the second yeast sequence. Phylogenetic trees were then inferred in the same way used in the phylome (see above), and duplication densities were calculated (see S13 Fig)

### Gene Order Information

Orthologous relationships between species and gene order data were obtained from the YGOB. Blocks of conserved synteny between the *S*. *cerevisiae* genome and the ancestral genome as predicted by Gordon et al. [11] were considered as conserved syntenic blocks.

### Comparison of Gene Order between ZT and KLE Genomes

The genome of *L*. *waltii* was not used because of the high fragmentation of the assembly. Genes in the genomes were arranged using *Z*. *rouxii* as reference (see S5 Table). Genomes were scanned for the presence of breaks in gene order that were common in the KLE clade and not found in either ZT species. Orthologs of the genes surrounding the breaks were searched in five post-WGD species (*S*. *cerevisiae*, *Tetrapisispora blattae*, *Kazachstania naganishii*, *Naumovozyma castellii*, and *C*. *glabrata*) in order to assess whether they followed the ZT or the KLE clade in their gene order (see S5 Table).

### Computation of the Number of Consecutive Pairs of Genes Conserved between Species

For each pair of genes located next to each other in the *S*. *cerevisiae* genome, we checked whether the orthologs in each of the ZT-KLE species were also contiguous. The same procedure was repeated in order to compare the ZT and KLE species.

### Computation of Number of Synteny Rearrangements

For each syntenic block, the orthologs were obtained for each of the seven species in the ZT and KLE clades. MGR [43] was used to compute the number of rearrangements that occurred between each ZT/KLE species and *S*. *cerevisiae*.

## Supporting Information

S1 Data Supporting data. (XLS)

S1 Fig Schematic representation of the duplication mapping process used in this work. (A) Graph representing the mapping of the phylome trees onto the species tree. Duplication events, as predicted by the species overlap algorithm, are marked as black dots. The loss of a gene in a given branch is marked in light grey. Duplications are mapped onto the species tree according to the lineages that diverged before and subsequent to it: A/B and C/E, respectively, for the tree in the top. (B) Representation of set 2, only ohnologous duplications are considered in this case. Grey trees represent phylome trees that were not used in this dataset, whereas black trees represent trees that contained ohnologous pairs. Red dots in the tree represent duplications that are considered since they give rise to the ohnologous pairs (red branches), while grey dots represent duplications that were ignored in this analysis.
(PDF)

S2 Fig Duplication densities calculated using the *V*. *polyspora* and *C*. *glabrata* phylomes. Duplication densities (average number of duplications per gene per branch) calculated using the *V*. *polyspora* (A) and *C*. *glabrata* (B) phylomes instead of the *S*. *cerevisiae* phylome. The left panel shows the species tree and the numbering of internal nodes for each analysis. The *x*-axis represents the different branches in the lineages of *C*. *glabrata* and *V*. *polyspora* as marked in the tree placed on the left of the figure. The *y*-axis represents duplication densities calculated for each branch. Lighter-coloured dots represent duplication densities for the whole phylome (set 1). Darker-coloured ones represent duplication rates using only trees that contain conserved ohnologs for the seed species and exclusively the node that gave rise to the duplication (set 2). Data on which this figure is based are provided in S1 Data.
(PDF)

S3 Fig Duplication densities calculated using a different species tree topology. Duplication densities (average number of duplications per gene per branch) calculated using a different species tree topology [26] in which the KLE group is not monophyletic. The *S*. *cerevisiae* phylome was used in this case. Figure representation is as in Fig 1. Data on which this figure is based are provided in S1 Data.
(PDF)

S4 Fig Duplication densities based on reconciliation. Duplication densities (average number of duplications per gene per branch) calculated using *S*. *cerevisiae* ohnologous gene trees inferred during phylome reconstruction. Duplication nodes were inferred using reconciliation as implemented in NOTUNG [27]. Mappings to the species tree were performed with the same program. Distribution of nodes is the same as in Fig 1. Data on which this figure is based are provided in S1 Data.
(PDF)

S5 Fig Duplication densities depending on sequence length. Duplication densities (average number of duplications per gene per branch) calculated for three groups of sequences of different lengths. Blue dots represent sequences shorter than 500 aa, green dots represent sequences between 500 aa and 1,000 aa, and yellow sequences represent sequences longer than 1,000 aa. Distribution of nodes is the same as in Fig 1. Data on which this figure is based are provided in S1 Data.
(PDF)

S6 Fig Number of trees that pass the support filter in different methods. Heat map representing the number of trees that pass the support filter in each pair of phylogenetic reconstruction methods. Numbers at the upper diagonal represent the number of trees that pass the support filter in the two methods. Numbers at the lower diagonal represent the number of trees that pass the filter in both methods and that agree on the prediction. Numbers at the diagonal represent the total number of trees that pass the filter for a given method. Background colours are graded according to the percentage of trees that pass the comparison compared to the available trees. Data on which this figure is based are provided in S1 Data.
(PDF)

S7 Fig Duplication densities for different phylogenetic methods. A–I: duplication densities (average number of duplications per gene per branch) calculated on a set of 846 trees with conserved ohnologous pairs. Graphs are drawn as in Fig 1. Details on each phylogenetic method can be found in S1 Table. J: number of trees with conserved ohnologs that pass the filters for each phylogenetic method. Coloured dots correlate with the colours used in the name tags of the different methods. Data on which this figure is based are provided in S1 Data.
(PDF)

S8 Fig Duplication densities for simulated data. Duplication densities (average number of duplications per gene per branch) calculated from the simulation of 5,160 trees in which an additional *S*. *cerevisiae* paralogous branch was placed. The branch length of one of the duplicates is 20 times longer than the other one. The *x*-axis represents the tree nodes of the *S*. *cerevisiae* lineage. The *y*-axis represents the duplication rate. Data on which this figure is based are provided in S1 Data.
(PDF)

S9 Fig Representative topologies found in reduced phylomes. Reduced phylomes contained only one seed species and one species from each of the ZT and KLE clades at the time, in order to simplify the analysis. Only relevant nodes with an aLRT support higher than 0.95 were used. (A) Trees depicting the three possible topologies considered. (B) Pie charts represent the percentage of trees in each phylome that support each of the topologies shown in A. The tag on top of each pie chart represents the combination of seed, ZT, and KLE species taken in the phylome. A: *A*. *gossypii*, C: *Candida glabrata*, K: *K*. *lactis*, Lk: *L*. *kluyveri*, Lt: *L*. *thermotolerans*, Lw: *L*. *waltii*, S: *S*. *cerevisiae*, T: *T*. *delbrueckii*, V: *Vanderwaltozyma polyspora*, Z: *Z*. *rouxii*. For example, STK is a combination of *S*. *cerevisiae*, *T*. *delbrueckii*, and *K*. *lactis*. Numbers indicate the amount of trees used in the analysis. Data on which this figure is based are provided in S1 Data.
(PDF)

S10 Fig Representative topologies found in reduced phylomes considering only ohnologs. Same as S9 Fig, but only trees containing conserved ohnologous pairs were considered. Data on which this figure is based are provided in S1 Data.
(PDF)

S11 Fig Representative topologies found in reduced phylomes reconstructed using different phylogenetic methods. For each phylogenetic method described in S1 Table, the first two pie charts represent the distribution of topologies found in the whole phylome (see S9 Fig). The third and fourth pie charts represent the same pie charts, but only for trees with conserved ohnologs (see S10 Fig). Data on which this figure is based are provided in S1 Data.
(PDF)

S12 Fig Representative topologies found in trees with conserved ohnologs. Topologies as predicted in the reduced phylomes in which conserved pairs of ohnologs were included. (A) Trees depicting the nine possible topologies considered. (B) Pie charts drawn for each combination of parental and seed species. Tags on top of each pie chart represent the combination of seed, ZT, and KLE species taken in the phylome as in S9 Fig. Data on which this figure is based are provided in S1 Data.
(PDF)

S13 Fig Duplication densities for simulated sets of gene conversion trees. (A) Species tree along which sequences were made to evolve. (B) Species tree used to map duplication densities (average number of duplications per gene per branch). (C) Duplication densities for sets of genes affected by different percentages of gene conversion.
(PDF)

S14 Fig Chronogram representing the estimated divergence times for fungi. The dot represents the point used to calibrate the tree. The red branch represents the minimal divergence between the two putative parental species at the moment of hybridization.
(PDF)

S1 Table List of phylogenetic methods used. Table listing the phylogenetic methods used to reconstruct a phylogenetic trees to test for consistency across methods. The first column indicates the name assigned to each method that can also be found in S6, S7, and S11 Figs. The second column indicates the program used. The third column indicates the evolutionary model used. The fourth column shows the topology search algorithm used, and the last column shows the method used to calculate branch support.
(DOCX)

S2 Table List of phylomes reconstructed or used in this study. The first column indicates the species used as seed to reconstruct the phylome. The second column indicates the phylome ID under which the phylome can be found at phylomeDB (http://phylomedb.org). The third and fourth columns indicate the species that were used as parental species when the phylomes were searched for topology distributions of their trees.
(DOCX)

S3 Table Table listing the reduced phylomes reconstructed in this study. For each phylome, the seed species and the two chosen parental species are listed. All trees can be found at http://genome.crg.es/~mmarcet/yeast_hybrids/phylome_table.htm.
(DOCX)

S4 Table Gene order conservation among ZT and KLE groups. Species are represented by two columns each: the first represents the protein code and the second the number of the position of the gene in the genome. Genes are coloured according to chromosome. Species are in the following order: *Z*. *rouxii*, which was used to order the other species, *T*. *delbrueckii*, *L*. *kluyveri*, *K*. *lactis*, *A*. *gossypii*, and *L*. *thermotolerans*. White cells indicate the loss of a gene in that position. Synteny breaks in the KLE group with respect to the ZT group are called by placing a tag in the last column.
(XLS)

S5 Table Representation of the 32 breaks in gene order in the ancestral KLE clade in the post-WGD species. For each break, indicated by an appropriate header, three groups of proteins are found. Each row represents orthologous genes, and each column represents one of the 12 species used. The ancestral genome predicted by Gordon et al. [11] is included. Post-WGDs are occupied by two columns each. For each break and each species, protein codes written in the same colour are found close to each other in the genome. Genes in black are genes that have no gene in close proximity within the set we are showing. For each break, the central set of genes, shown by five or six rows of genes, indicates the break in gene order that can be seen marked with a thick black line at the KLE columns (last four columns). At the break point, two proteins can be found at either side of the break. The genes located at either side of these genes can be found partly in the central set of genes and partly in the upper and lower set of genes. Empty rows indicate missing orthologs. Orthologs for the post-WGD were arranged in such a way as to maximize gene order conservation.
(XLS)

S6 Table Gene order conservation of pairs of genes between yeast and different species from putative parental clades. The total number of yeasts pairs considered is 6,961. The first two columns indicate the species name and whether it belongs to the ZT or the KLE clade. The third column indicates pairs of genes in *S*. *cerevisiae* whose orthologs in the parental species are also found together in the genome. Column four indicates the percentage of pairs found conserved. Column number five indicates pairs of genes whose gene order was conserved uniquely between *S*. *cerevisiae* and a given parent. The last column indicates the number of pairs whose gene order was conserved exclusively between *S*. *cerevisiae* and the ZT-KLE species.
(DOCX)

S7 Table Gene order conservation of pairs of genes between ZT and KLE species. The first two columns indicate the two parents used in the analysis. The third column indicates the number of pairs of genes placed consecutively in a ZT species whose orthologs in a KLE are also placed together in the genome. Column four indicates the percentage of pairs that column three represents. The final column indicates the orthologs in KLE that do not conserve gene order when compared to ZT.
(DOCX)

S8 Table List of proteomes used in the main phylomes. The first two columns indicate, in this order, species name and data source. The two additional columns represent the number of times the genome was used to reconstruct complete phylomes and reduced phylomes, respectively. Asterisks denote that the species was used as seed. Sources listed: Genolevures: http://www.genolevures.org/; Joint Genome Institute (JGI): http://www.jgi.doe.gov/; National Center for Biotechnology Information (NCBI): http://www.ncbi.nlm.nih.gov/genbank/; Quest for Orthologs: http://questfororthologs.org/; UniProt: http://www.uniprot.org/; and YGOB: http://ygob.ucd.ie/.
(DOCX)

S9 Table List of proteomes used in additional phylomes. The first two columns indicate, in this order, species name and data source. The third column contains the ID of the phylome a species was used in. See S2 Table for phylomeDB ID correspondence. Asterisks denote the species was used as seed in the phylome. Additional sources listed: Broad Institute: http://www.broadinstitute.org/; Hyphal Tip: http://fungalgenomes.org/blog/available-genomes/; *Saccharomyces* Genome Database (SGD): http://www.yeastgenome.org/.
(DOCX)

## Abbreviations

aLRT: approximate likelihood ratio test
BI: Bayesian inference
JGI: Joint Genome Institute
KLE: *Kluyveromyces*, *Lachancea*, and *Eremothecium*
LBA: long-branch attraction
MGR: Multiple Genome Rearrangements
ML: maximum likelihood
NCBI: National Center for Biotechnology Information
NJ: neighbour joining
NNI: nearest-neighbour interchange
SGD: *Saccharomyces* Genome Database
SH: Shimodaira–Hasegawa
SPR: subtree pruning and regrafting
WGD: whole-genome duplication
YGOB: Yeast Gene Order Browser
