# Supplementary material for: Beyond the Whole-Genome Duplication: Phylogenetic Evidence for an Ancient Interspecies Hybridization in the Baker's Yeast Lineage
Source: PLoS Biol. 2015 Aug 7;13(8):e1002220. doi: 10.1371/journal.pbio.1002220 (PMC4529251; doi:10.1371/journal.pbio.1002220)
Supplement: S1 Fig — (A) Graph representing the mapping of the phylome trees onto the species tree. Duplication events, as predicted by the species overlap algorithm, are marked as black dots. The loss of a gene in a given branch is marked in light grey. Duplications are mapped onto the species tree according to the lineages that diverged before and subsequent to it: A/B and C/E, respectively, for the tree in the top. (B) Representation of set 2, only ohnologous duplications are considered in this case. Grey trees represent phylome trees that were not used in this dataset, whereas black trees represent trees that contained ohnologous pairs. Red dots in the tree represent duplications that are considered since they give rise to the ohnologous pairs (red branches), while grey dots represent duplications that were ignored in this analysis. (PDF) [file pbio.1002220.s002.pdf]

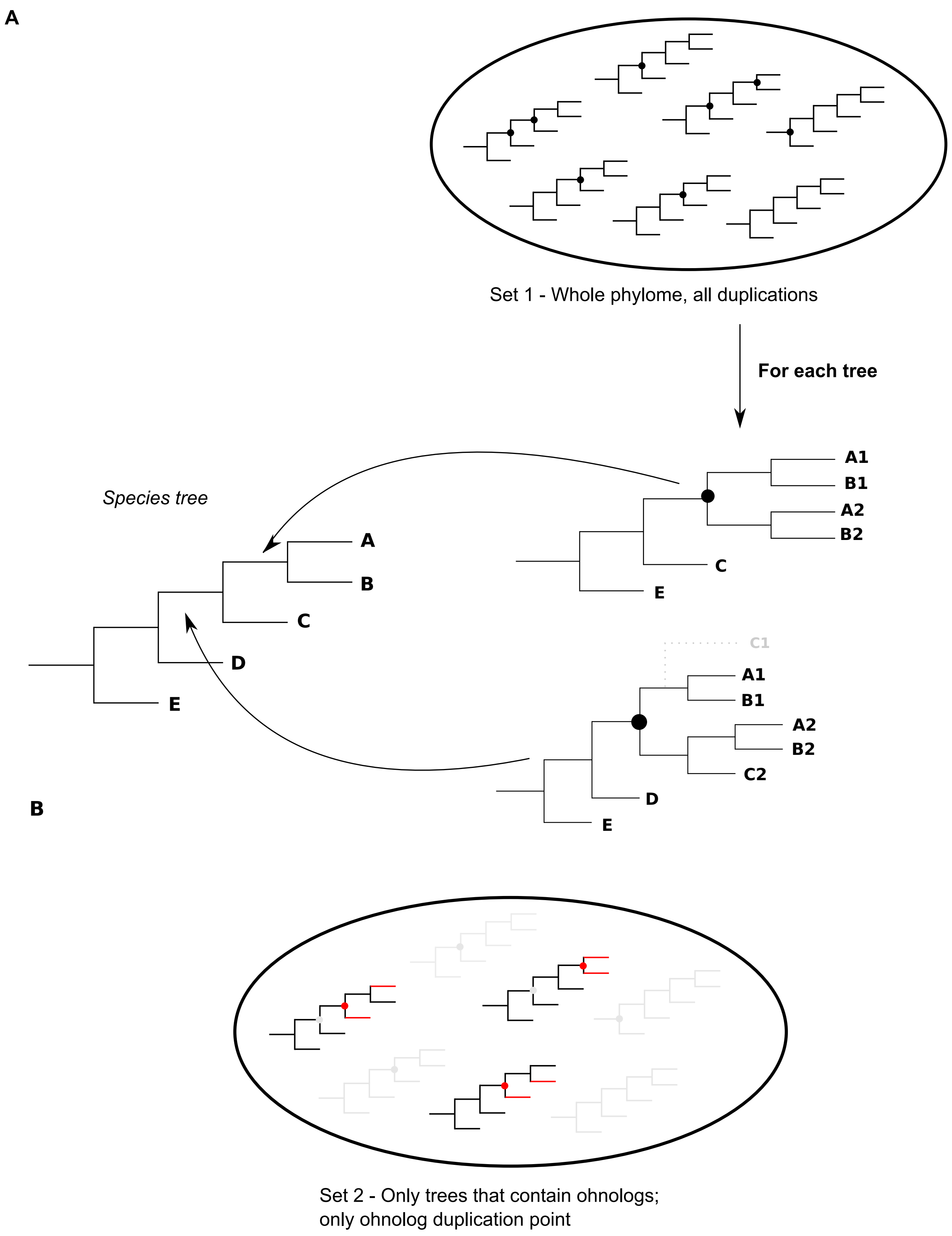
A
Set 1 - Whole phylome, all duplications
For each tree
Species tree
A
B
C
D
E
A1
B1
A2
B2
C
E
C1
A1
B1
A2
B2
C2
D
E
B
Set 2 - Only trees that contain ohnologs;
only ohnolog duplication point
