# Supplementary figures and images for: Beyond the Whole-Genome Duplication: Phylogenetic Evidence for an Ancient Interspecies Hybridization in the Baker's Yeast Lineage
Source: PLoS Biol. 2015 Aug 7;13(8):e1002220. doi: 10.1371/journal.pbio.1002220 (PMC4529251; doi:10.1371/journal.pbio.1002220)

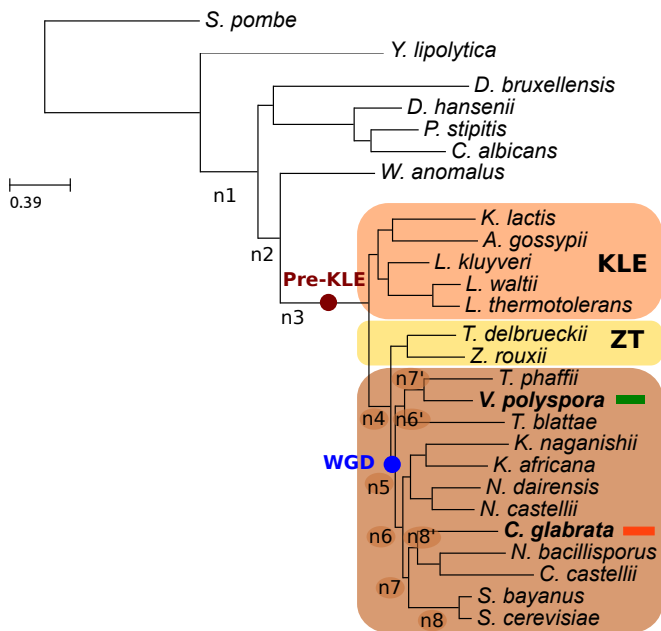

A

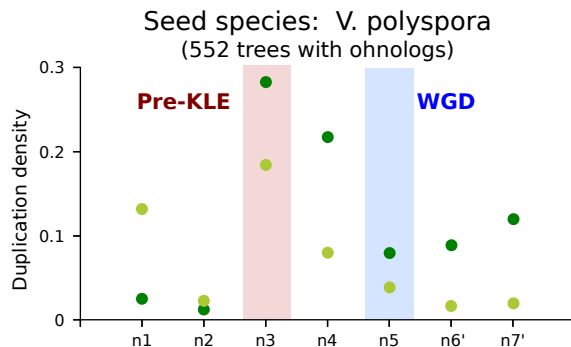

B

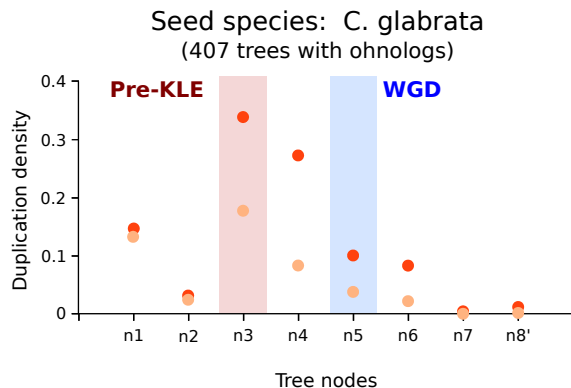

Supplement: S2 Fig — Duplication densities (average number of duplications per gene per branch) calculated using the V. polyspora (A) and C. glabrata (B) phylomes instead of the S. cerevisiae phylome. The left panel shows the species tree and the numbering of internal nodes for each analysis. The x-axis represents the different branches in the lineages of C. glabrata and V. polyspora as marked in the tree placed on the left of the figure. The y-axis represents duplication densities calculated for each branch. Lighter-coloured dots represent duplication densities for the whole phylome (set 1). Darker-coloured ones represent duplication rates using only trees that contain conserved ohnologs for the seed species and exclusively the node that gave rise to the duplication (set 2). Data on which this figure is based are provided in S1 Data. (PDF) [file pbio.1002220.s003.pdf]

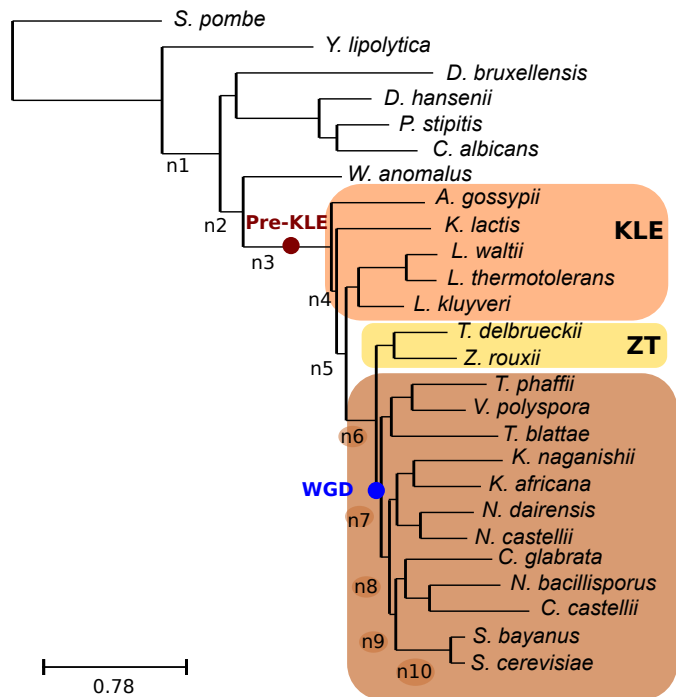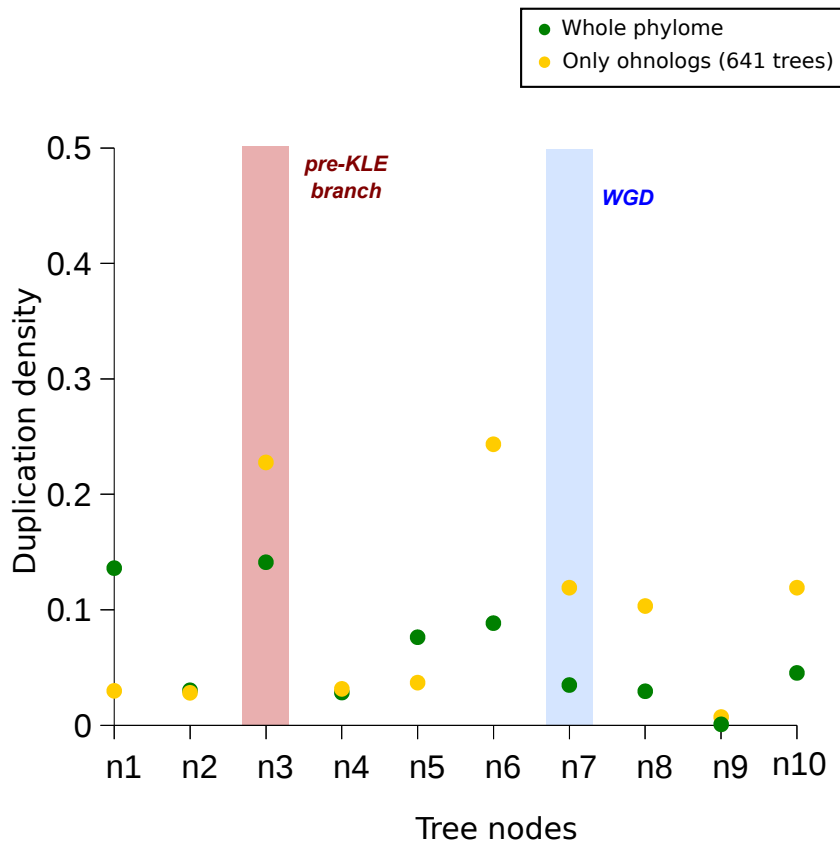

Supplement: S3 Fig — Duplication densities (average number of duplications per gene per branch) calculated using a different species tree topology [26] in which the KLE group is not monophyletic. The S. cerevisiae phylome was used in this case. Figure representation is as in Fig 1. Data on which this figure is based are provided in S1 Data. (PDF) [file pbio.1002220.s004.pdf]

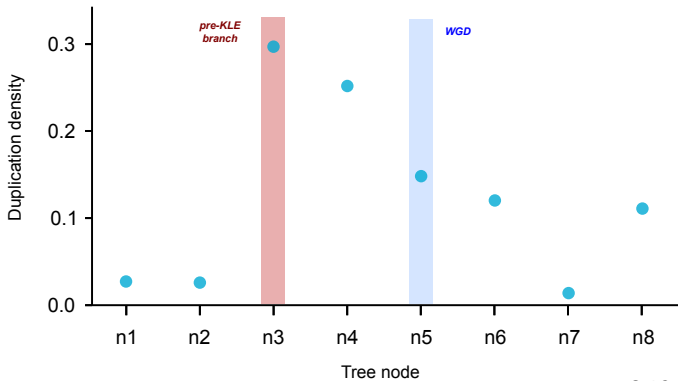

846 trees

Supplement: S4 Fig — Duplication densities (average number of duplications per gene per branch) calculated using S. cerevisiae ohnologous gene trees inferred during phylome reconstruction. Duplication nodes were inferred using reconciliation as implemented in NOTUNG [27]. Mappings to the species tree were performed with the same program. Distribution of nodes is the same as in Fig 1. Data on which this figure is based are provided in S1 Data. (PDF) [file pbio.1002220.s005.pdf]

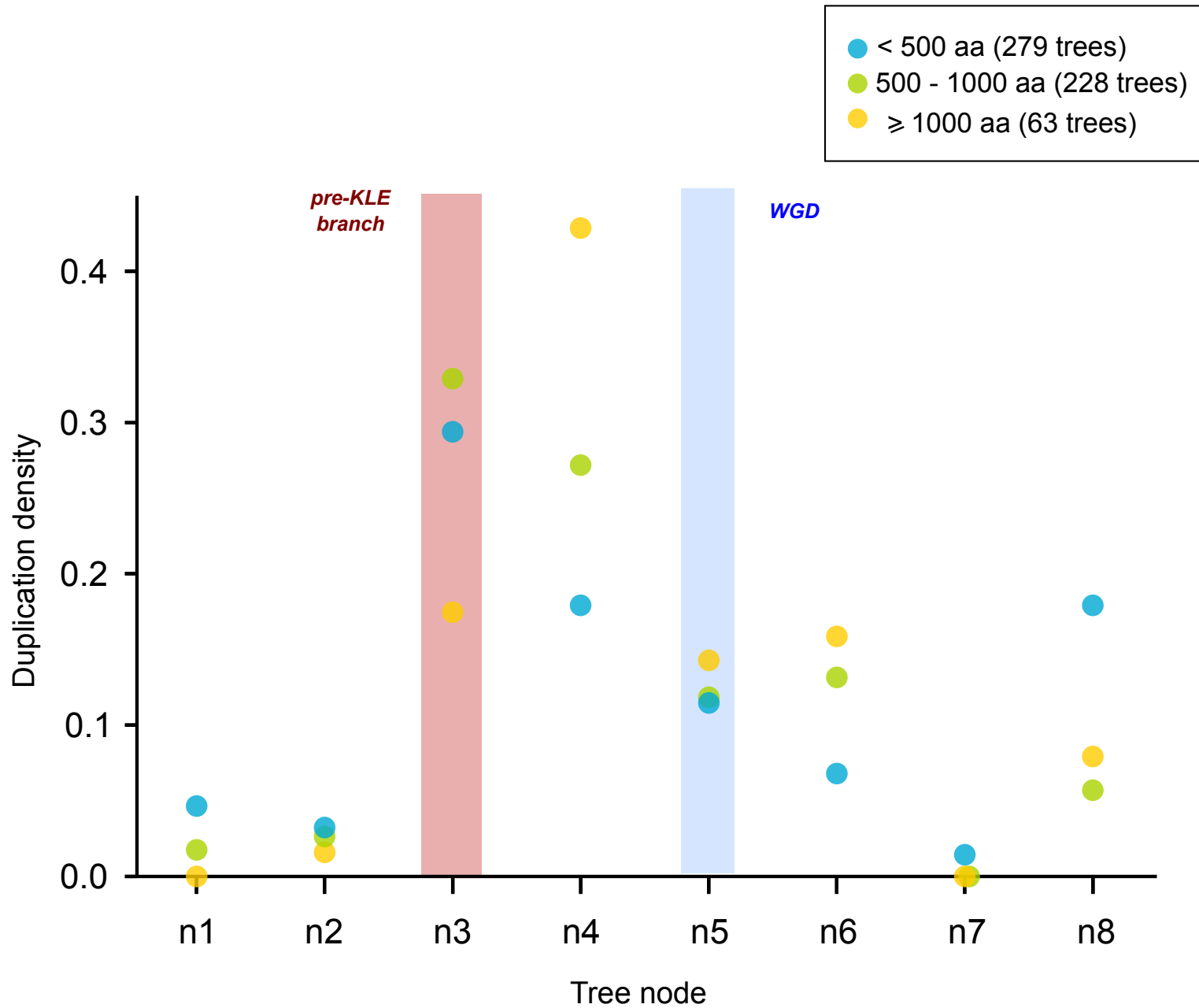

Supplement: S5 Fig — Duplication densities (average number of duplications per gene per branch) calculated for three groups of sequences of different lengths. Blue dots represent sequences shorter than 500 aa, green dots represent sequences between 500 aa and 1,000 aa, and yellow sequences represent sequences longer than 1,000 aa. Distribution of nodes is the same as in Fig 1. Data on which this figure is based are provided in S1 Data. (PDF) [file pbio.1002220.s006.pdf]

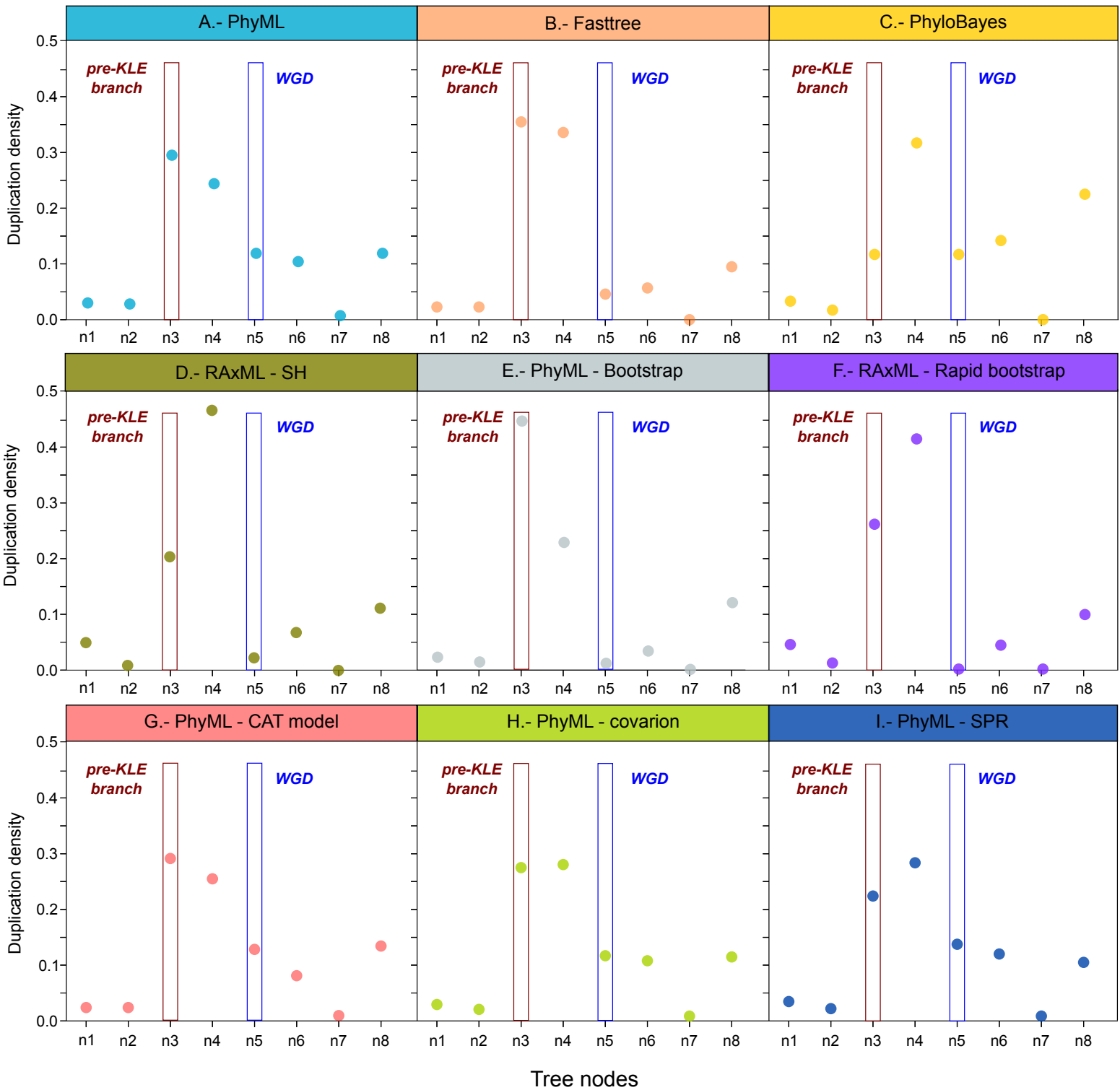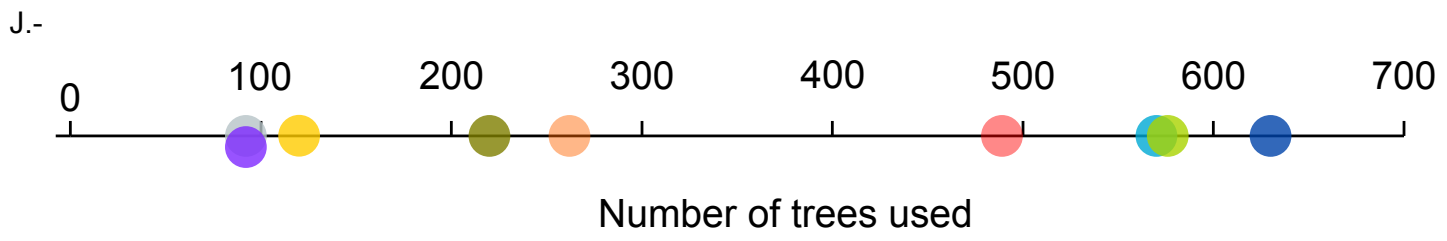

Supplement: S7 Fig — A–I: duplication densities (average number of duplications per gene per branch) calculated on a set of 846 trees with conserved ohnologous pairs. Graphs are drawn as in Fig 1. Details on each phylogenetic method can be found in S1 Table. J: number of trees with conserved ohnologs that pass the filters for each phylogenetic method. Coloured dots correlate with the colours used in the name tags of the different methods. Data on which this figure is based are provided in S1 Data. (PDF) [file pbio.1002220.s008.pdf]

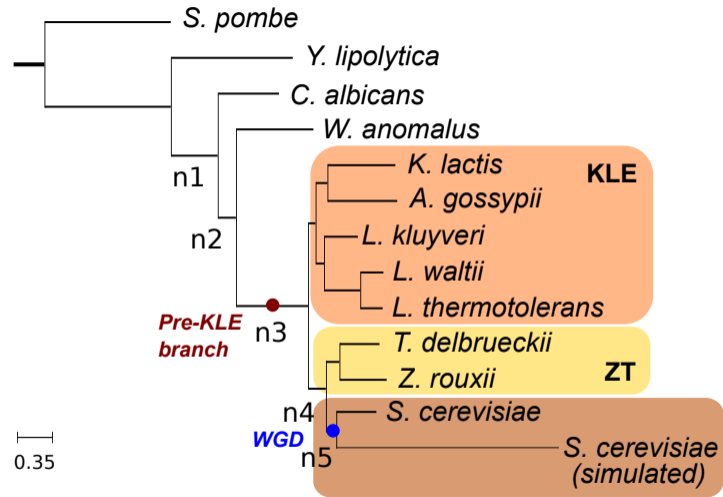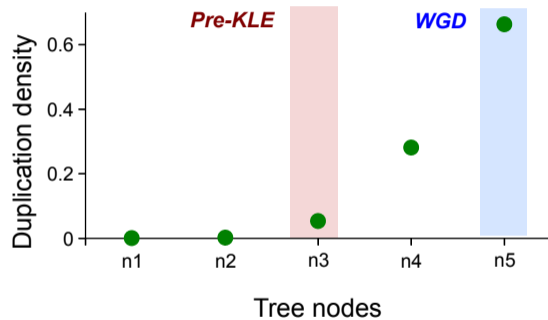

Supplement: S8 Fig — Duplication densities (average number of duplications per gene per branch) calculated from the simulation of 5,160 trees in which an additional S. cerevisiae paralogous branch was placed. The branch length of one of the duplicates is 20 times longer than the other one. The x-axis represents the tree nodes of the S. cerevisiae lineage. The y-axis represents the duplication rate. Data on which this figure is based are provided in S1 Data. (PDF) [file pbio.1002220.s009.pdf]

a)

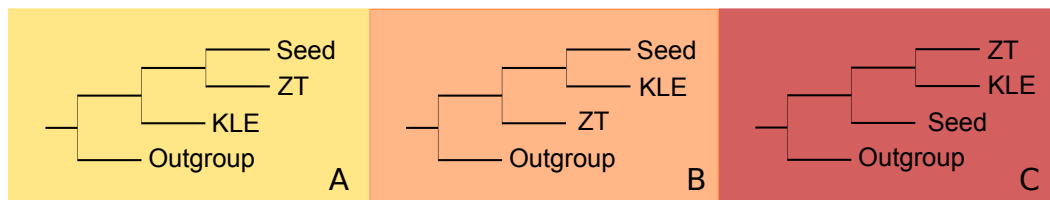

b)

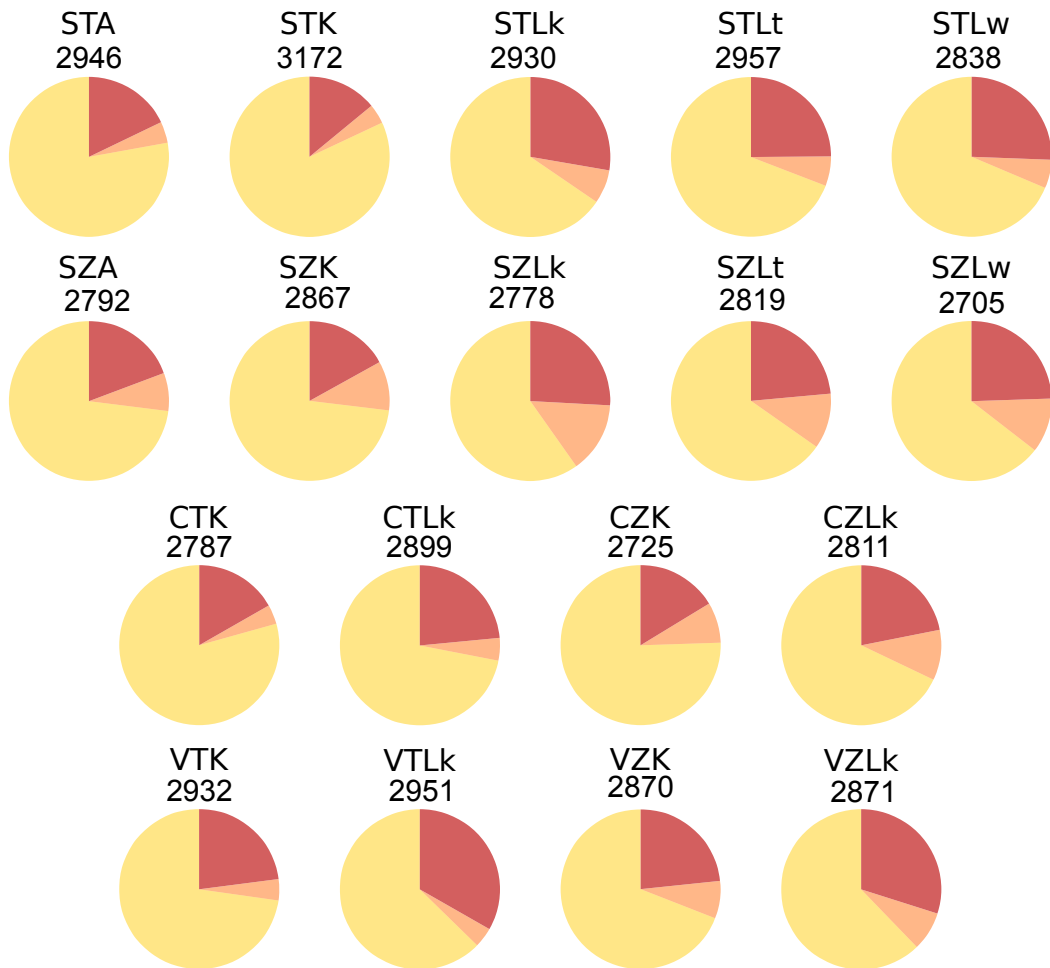

Supplement: S9 Fig — Reduced phylomes contained only one seed species and one species from each of the ZT and KLE clades at the time, in order to simplify the analysis. Only relevant nodes with an aLRT support higher than 0.95 were used. (A) Trees depicting the three possible topologies considered. (B) Pie charts represent the percentage of trees in each phylome that support each of the topologies shown in A. The tag on top of each pie chart represents the combination of seed, ZT, and KLE species taken in the phylome. A: A. gossypii, C: Candida glabrata, K: K. lactis, Lk: L. kluyveri, Lt: L. thermotolerans, Lw: L. waltii, S: S. cerevisiae, T: T. delbrueckii, V: Vanderwaltozyma polyspora, Z: Z. rouxii. For example, STK is a combination of S. cerevisiae, T. delbrueckii, and K. lactis. Numbers indicate the amount of trees used in the analysis. Data on which this figure is based are provided in S1 Data. (PDF) [file pbio.1002220.s010.pdf]

a)

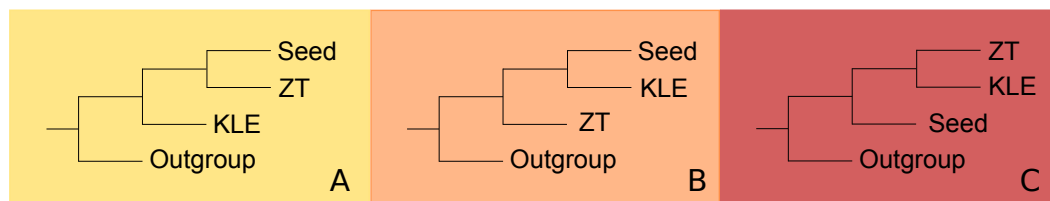

b)

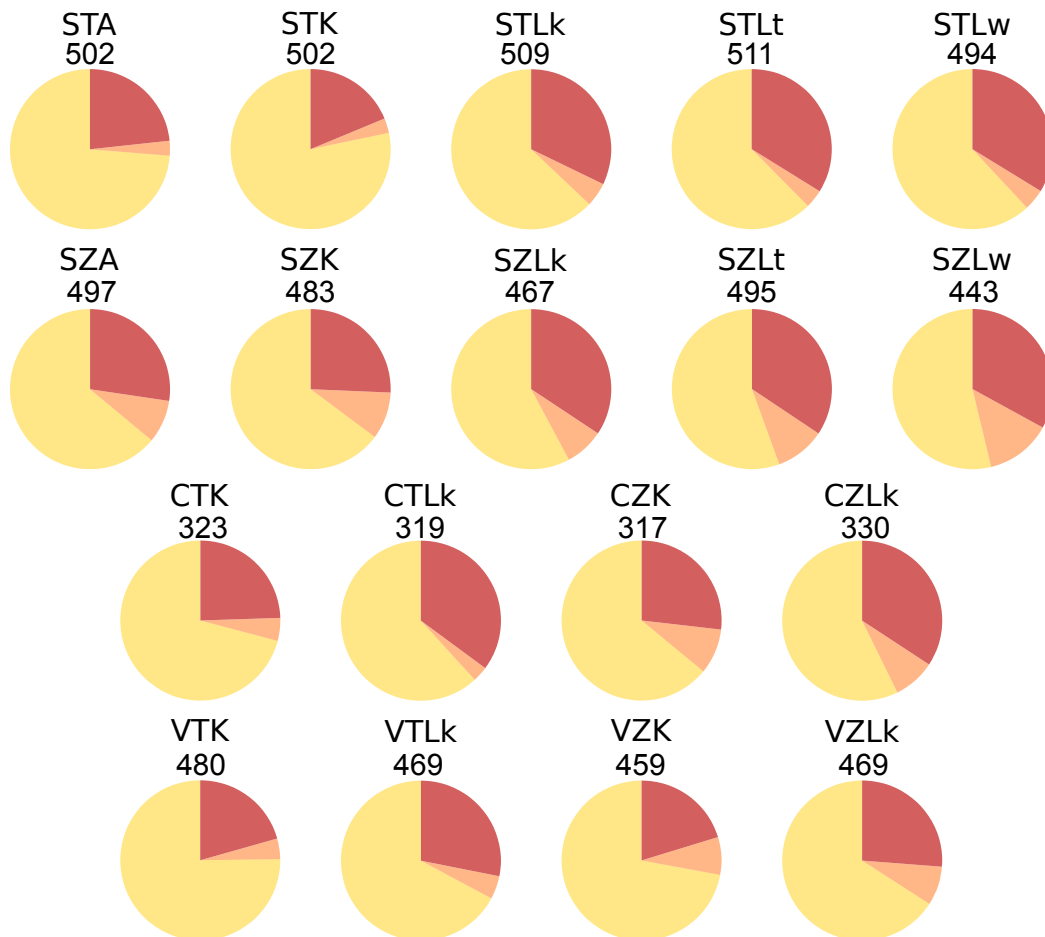

Supplement: S10 Fig — Same as S9 Fig, but only trees containing conserved ohnologous pairs were considered. Data on which this figure is based are provided in S1 Data. (PDF) [file pbio.1002220.s011.pdf]

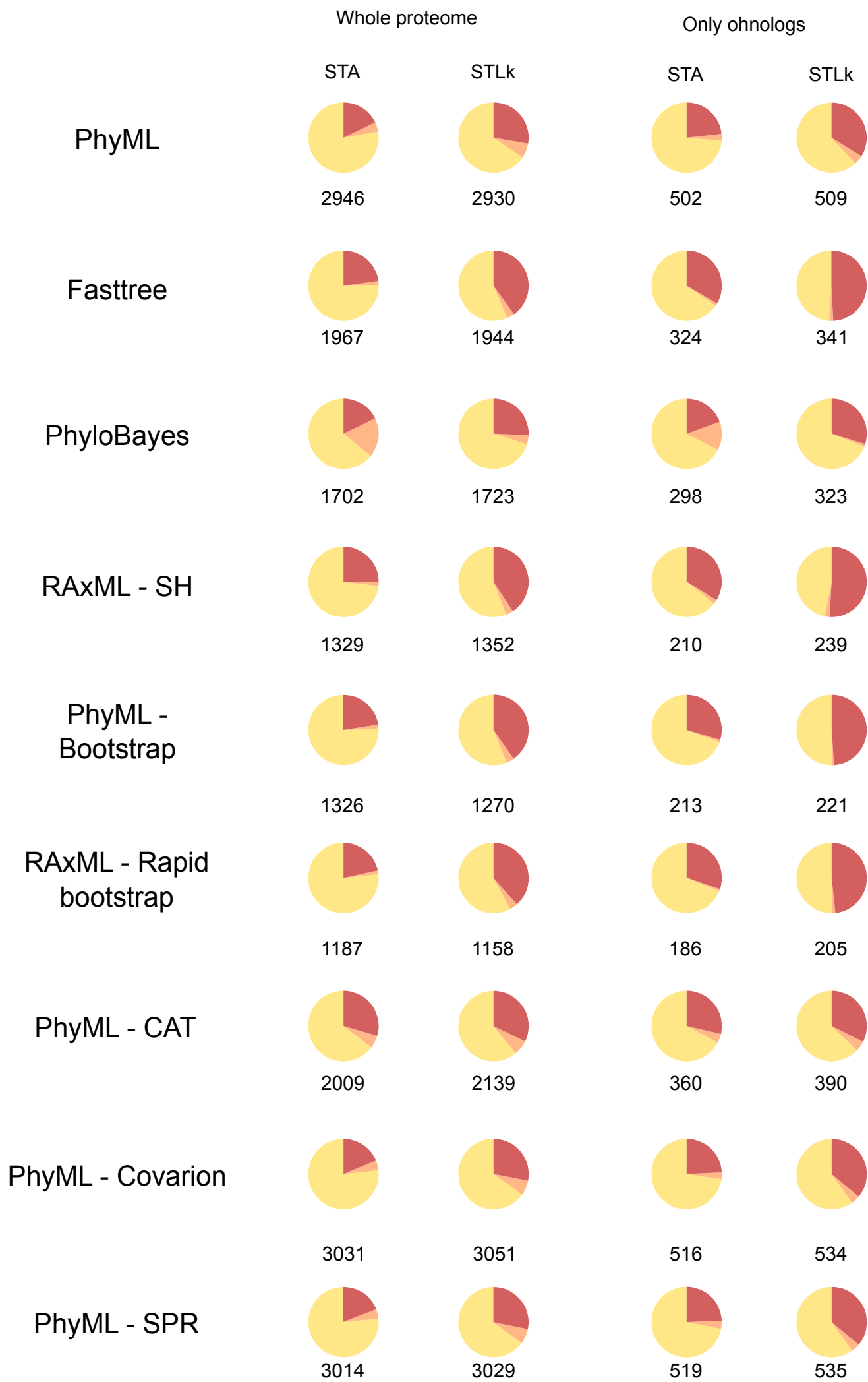

Supplement: S11 Fig — For each phylogenetic method described in S1 Table, the first two pie charts represent the distribution of topologies found in the whole phylome (see S9 Fig). The third and fourth pie charts represent the same pie charts, but only for trees with conserved ohnologs (see S10 Fig). Data on which this figure is based are provided in S1 Data. (PDF) [file pbio.1002220.s012.pdf]

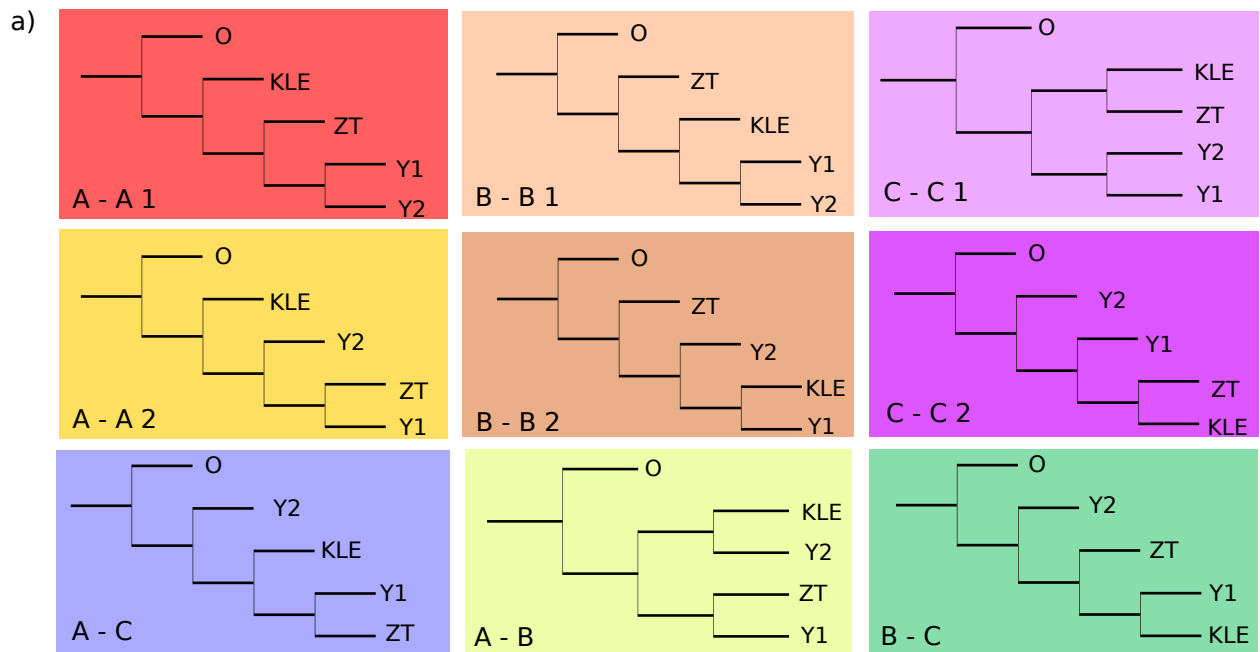

b)

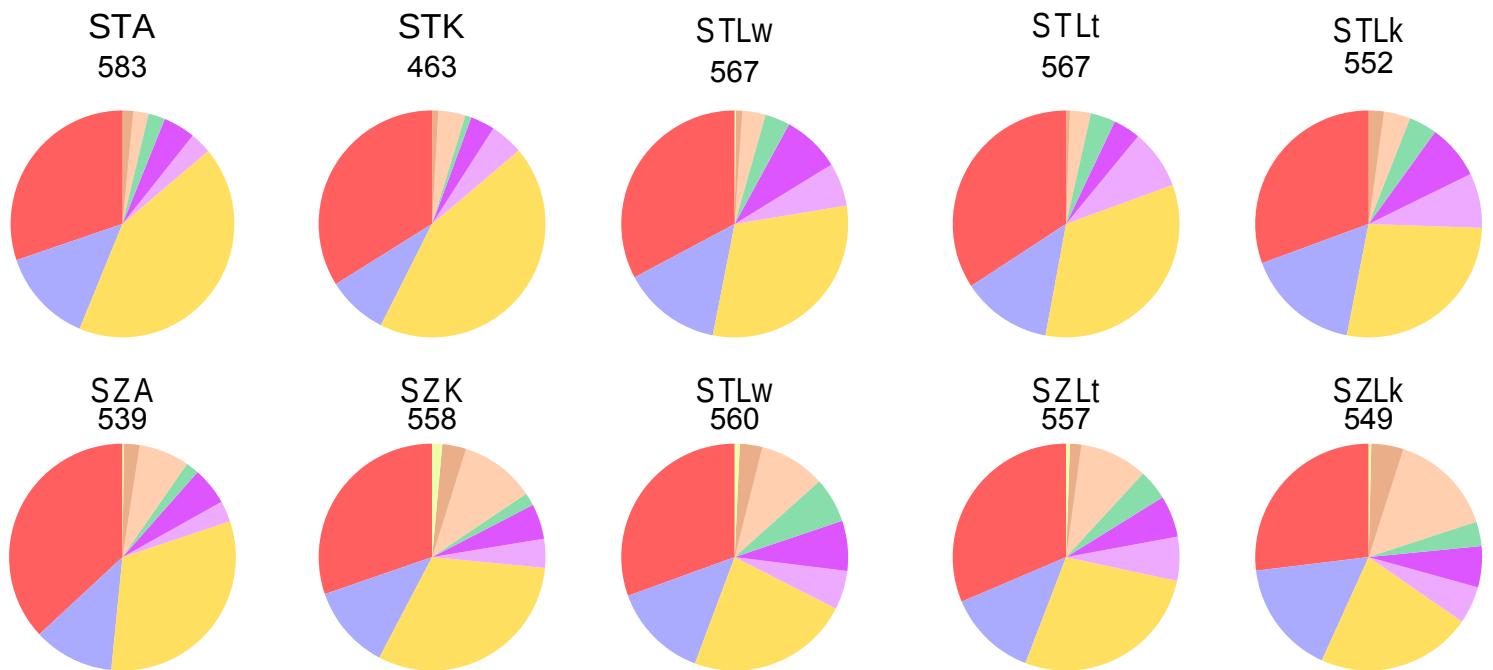

Supplement: S12 Fig — Topologies as predicted in the reduced phylomes in which conserved pairs of ohnologs were included. (A) Trees depicting the nine possible topologies considered. (B) Pie charts drawn for each combination of parental and seed species. Tags on top of each pie chart represent the combination of seed, ZT, and KLE species taken in the phylome as in S9 Fig. Data on which this figure is based are provided in S1 Data. (PDF) [file pbio.1002220.s013.pdf]

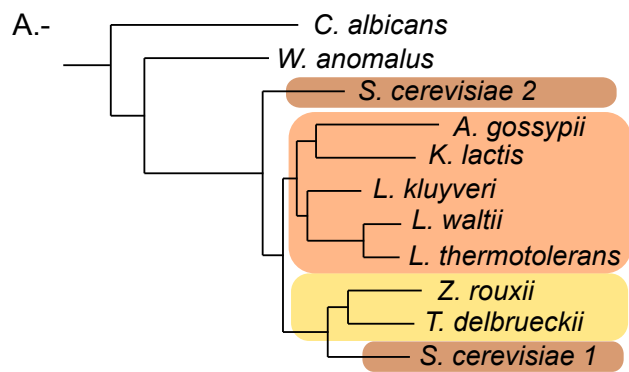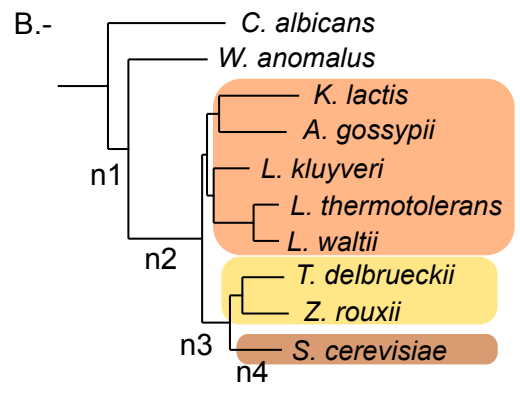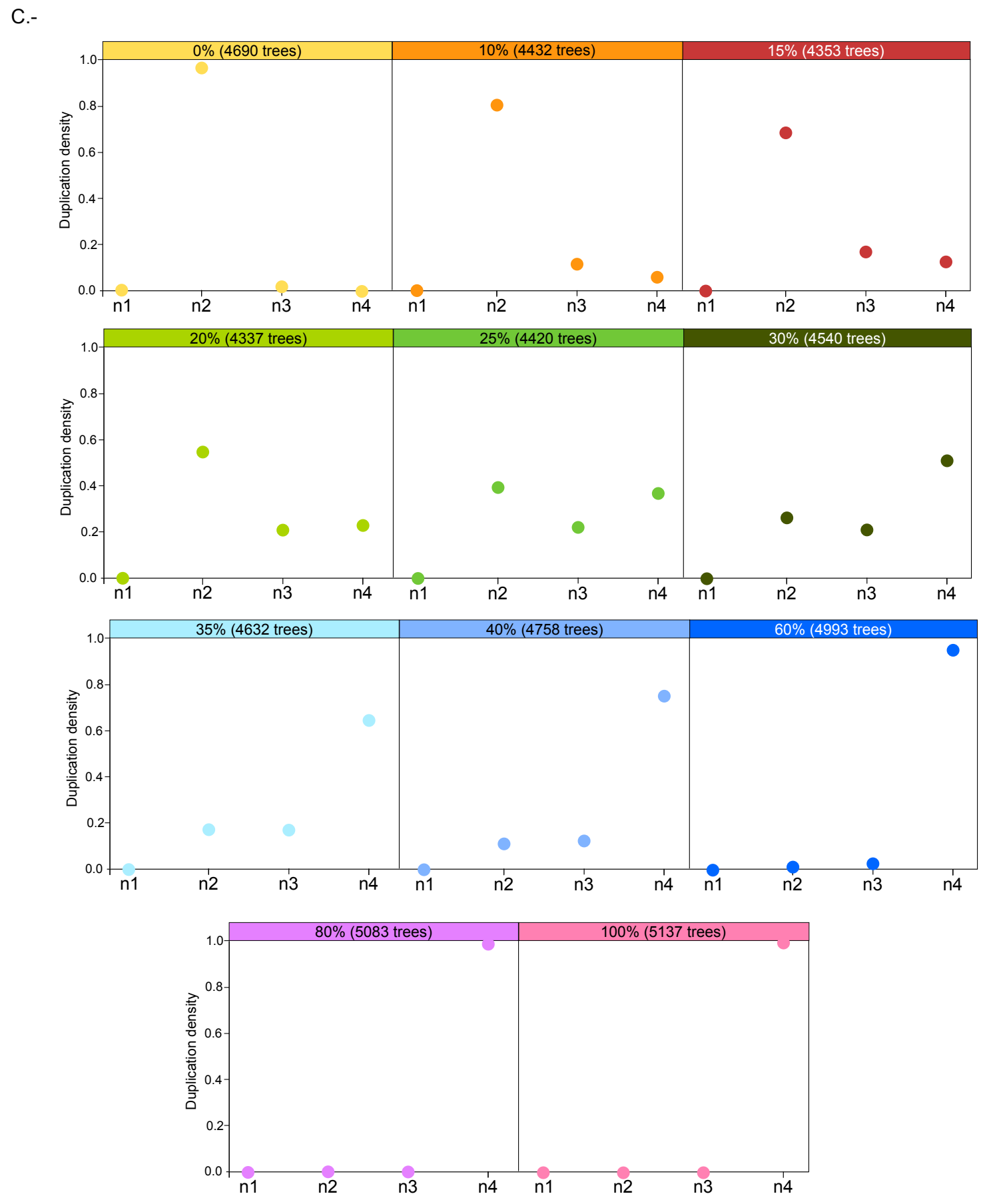

Supplement: S13 Fig — (A) Species tree along which sequences were made to evolve. (B) Species tree used to map duplication densities (average number of duplications per gene per branch). (C) Duplication densities for sets of genes affected by different percentages of gene conversion. (PDF) [file pbio.1002220.s014.pdf]

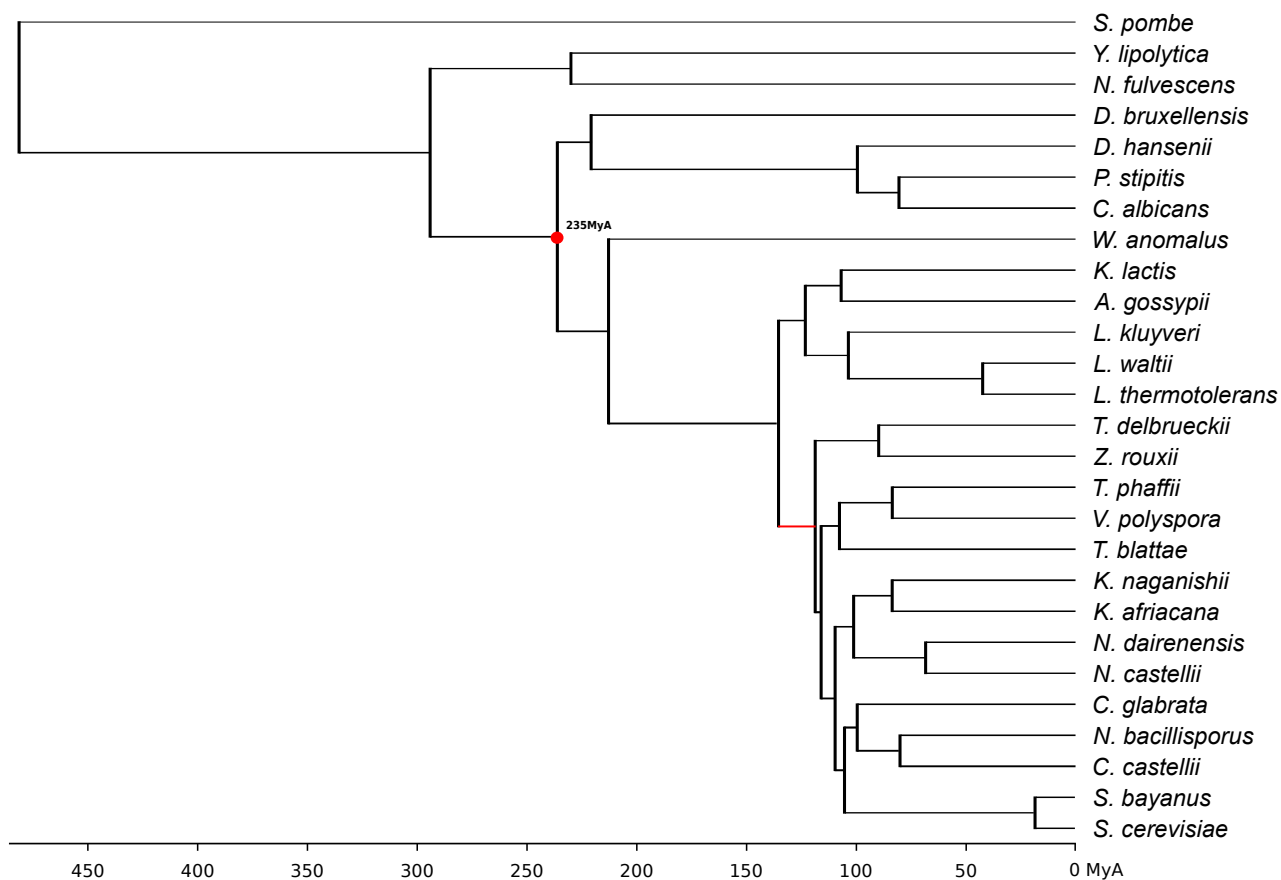

Supplement: S14 Fig — The dot represents the point used to calibrate the tree. The red branch represents the minimal divergence between the two putative parental species at the moment of hybridization. (PDF) [file pbio.1002220.s015.pdf]
