# Supplementary material for: Beyond the Whole-Genome Duplication: Phylogenetic Evidence for an Ancient Interspecies Hybridization in the Baker's Yeast Lineage
Source: PLoS Biol. 2015 Aug 7;13(8):e1002220. doi: 10.1371/journal.pbio.1002220 (PMC4529251; doi:10.1371/journal.pbio.1002220)
Supplement: S6 Fig — Heat map representing the number of trees that pass the support filter in each pair of phylogenetic reconstruction methods. Numbers at the upper diagonal represent the number of trees that pass the support filter in the two methods. Numbers at the lower diagonal represent the number of trees that pass the filter in both methods and that agree on the prediction. Numbers at the diagonal represent the total number of trees that pass the filter for a given method. Background colours are graded according to the percentage of trees that pass the comparison compared to the available trees. Data on which this figure is based are provided in S1 Data. (PDF) [file pbio.1002220.s007.pdf]

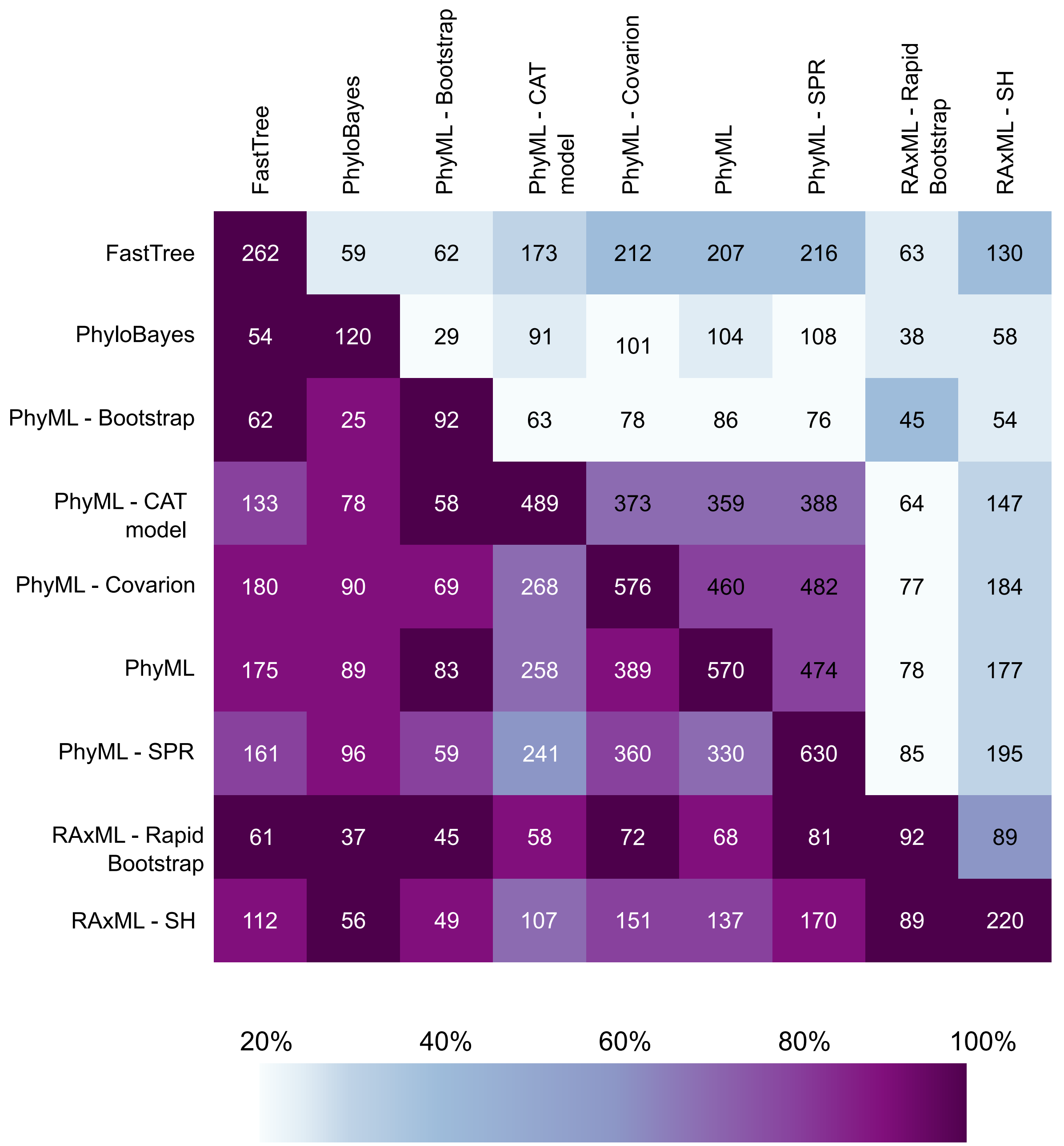
FastTree
PhyloBayes
PhyML - Bootstrap
PhyML - CAT model
PhyML - Covarion
PhyML
PhyML - SPR
RAxML - Rapid Bootstrap
RAxML - SH
FastTree 262 59 62 173 212 207 216 63 130
PhyloBayes 54 120 29 91 101 104 108 38 58
PhyML - Bootstrap 62 25 92 63 78 86 76 45 54
PhyML - CAT model 133 78 58 489 373 359 388 64 147
PhyML - Covarion 180 90 69 268 576 460 482 77 184
PhyML 175 89 83 258 389 570 474 78 177
PhyML - SPR 161 96 59 241 360 330 630 85 195
RAxML - Rapid Bootstrap 61 37 45 58 72 68 81 92 89
RAxML - SH 112 56 49 107 151 137 170 89 220
20%
40%
60%
80%
100%
