# Supplementary material for: Beyond the Whole-Genome Duplication: Phylogenetic Evidence for an Ancient Interspecies Hybridization in the Baker's Yeast Lineage
Source: PLoS Biol. 2015 Aug 7;13(8):e1002220. doi: 10.1371/journal.pbio.1002220 (PMC4529251; doi:10.1371/journal.pbio.1002220)
Supplement: S1 Table — Table listing the phylogenetic methods used to reconstruct a phylogenetic trees to test for consistency across methods. The first column indicates the name assigned to each method that can also be found in S6, S7, and S11 Figs. The second column indicates the program used. The third column indicates the evolutionary model used. The fourth column shows the topology search algorithm used, and the last column shows the method used to calculate branch support. (DOCX) [file pbio.1002220.s016.docx]

**S1 Table:**  List of phylogenetic methods used.

| Name | Program | Evolutionary method | Topology search algorithm | Support |
| --- | --- | --- | --- | --- |
| Fasttree | Fasttree | JTT+CAT | NNI + SPR | SH-like |
| PhyML | PhyML v3 | Best fitting model | NNI | aLRT |
| PhyML – SPR | PhyML v3 | Best fitting model | SPR | aLRT |
| PhyML – Bootstrap | PhyML v3 | Best fitting model | NNI | Bootstrap |
| PhyML - COVARION | PhyML v3 | Best fitting model + COVARION | NNI | aLRT |
| PhyML – CAT model | PhyML - CAT | C20 | NNI | aLRT |
| RaxML – Rapid Bootstrap | RAxML HPC-SSE3 | LG | SPR | Rapid bootstrap |
| RaxML - SH | RAxML HPC-SSE3 | LG | SPR | SH |
| PhyloBayes | PhyloBayes v3.3f | CAT | - | Posterior probabilities |
