# Supplementary material for: Beyond the Whole-Genome Duplication: Phylogenetic Evidence for an Ancient Interspecies Hybridization in the Baker's Yeast Lineage
Source: PLoS Biol. 2015 Aug 7;13(8):e1002220. doi: 10.1371/journal.pbio.1002220 (PMC4529251; doi:10.1371/journal.pbio.1002220)
Supplement: S2 Table — The first column indicates the species used as seed to reconstruct the phylome. The second column indicates the phylome ID under which the phylome can be found at phylomeDB (http://phylomedb.org). The third and fourth columns indicate the species that were used as parental species when the phylomes were searched for topology distributions of their trees. (DOCX) [file pbio.1002220.s017.docx]

**S2 Table:**  List of phylomes reconstructed or used in this study

| Species name | Phylome ID | Parental A | Parental B |
| --- | --- | --- | --- |
| *Candida albicans* | 23 | *Candida dubliniensis* | *Candida tropicalis* |
| *Candida glabrata* | 207 | *Torulaspora delbrueckii; Zygosaccharomyces rouxii* | *Ashbya gossypii; Kluyveromyces lactis; Lachancea kluyveri; Lachancea thermotolerans; Lachancea waltii* |
| *Hortaea werneckii* | 211 | *Zymoseptoria tritici* | *Macrophomina phaseolina* |
| *Penicillium digitatum* | 150 | *Penicillium chrysogenum* | *Emericella nidulans* |
| *Rhizopus delemar* | 252 | *Mucor circinelloides* | *Phycomyces blakesleeanus* |
| *Saccharomyces cerevisiae* | 206 | *Torulaspora delbrueckii; Zygosaccharomyces rouxii* | *Ashbya gossypii; Kluyveromyces lactis; Lachancea kluyveri; Lachancea thermotolerans; Lachancea waltii* |
| *Saccharomyces cerevisiae x Saccharomyces kudriavzevii VIN7* | 210 | *Saccharomyces cerevisiae* | *Saccharomyces kudriavzevii* |
| *Saccharomyces pastorianus* | 209 | *Saccharomyces cerevisiae* | *Saccharomyces bayanus* |
| *Vanderwaltozyma polyspora* | 208 | *Torulaspora delbrueckii; Zygosaccharomyces rouxii* | *Ashbya gossypii; Kluyveromyces lactis; Lachancea kluyveri; Lachancea thermotolerans; Lachancea waltii* |
| *Saccharomyces cerevisiae*  *(Simulation)* | <http://genome.crg.es/~mmarcet/yeast_hybrids/phylome_table.htm> | *Torulaspora delbrueckii; Zygosaccharomyces rouxii* | *Ashbya gossypii; Kluyveromyces lactis; Lachancea kluyveri; Lachancea thermotolerans; Lachancea waltii* |
