# Supplementary material for: Beyond the Whole-Genome Duplication: Phylogenetic Evidence for an Ancient Interspecies Hybridization in the Baker's Yeast Lineage
Source: PLoS Biol. 2015 Aug 7;13(8):e1002220. doi: 10.1371/journal.pbio.1002220 (PMC4529251; doi:10.1371/journal.pbio.1002220)
Supplement: S3 Table — For each phylome, the seed species and the two chosen parental species are listed. All trees can be found at http://genome.crg.es/~mmarcet/yeast_hybrids/phylome_table.htm. (DOCX) [file pbio.1002220.s018.docx]

**S3 Table:** Table listing the reduced phylomes reconstructed in this study.

| Seed species | ZT | KLE |
| --- | --- | --- |
| *Saccharomyces cerevisiae* | *Torulaspora delbrueckii* | *Ashbya gossypii* |
| *Saccharomyces cerevisiae* | *Torulaspora delbrueckii* | *Kluyveromyces lactis* |
| *Saccharomyces cerevisiae* | *Torulaspora delbrueckii* | *Lachancea kluyveri* |
| *Saccharomyces cerevisiae* | *Torulaspora delbrueckii* | *Lachancea thermotolerans* |
| *Saccharomyces cerevisiae* | *Torulaspora delbrueckii* | *Lachancea waltii* |
| *Saccharomyces cerevisiae* | *Zygosaccharomyces rouxii* | *Ashbya gossypii* |
| *Saccharomyces cerevisiae* | *Zygosaccharomyces rouxii* | *Kluyveromyces lactis* |
| *Saccharomyces cerevisiae* | *Zygosaccharomyces rouxii* | *Lachancea kluyveri* |
| *Saccharomyces cerevisiae* | *Zygosaccharomyces rouxii* | *Lachancea thermotolerans* |
| *Saccharomyces cerevisiae* | *Zygosaccharomyces rouxii* | *Lachancea waltii* |
| *Candida glabrata* | *Torulaspora delbrueckii* | *Kluyveromyces lactis* |
| *Candida glabrata* | *Torulaspora delbrueckii* | *Lachancea kluyveri* |
| *Candida glabrata* | *Zygosaccharomyces rouxii* | *Kluyveromyces lactis* |
| *Candida glabrata* | *Zygosaccharomyces rouxii* | *Lachancea kluyveri* |
| *Vanderwaltozyma polyspora* | *Torulaspora delbrueckii* | *Kluyveromyces lactis* |
| *Vanderwaltozyma polyspora* | *Torulaspora delbrueckii* | *Lachancea kluyveri* |
| *Vanderwaltozyma polyspora* | *Zygosaccharomyces rouxii* | *Kluyveromyces lactis* |
| *Vanderwaltozyma polyspora* | *Zygosaccharomyces rouxii* | *Lachancea kluyveri* |
