# Supplementary material for: Beyond the Whole-Genome Duplication: Phylogenetic Evidence for an Ancient Interspecies Hybridization in the Baker's Yeast Lineage
Source: PLoS Biol. 2015 Aug 7;13(8):e1002220. doi: 10.1371/journal.pbio.1002220 (PMC4529251; doi:10.1371/journal.pbio.1002220)
Supplement: S6 Table — The total number of yeasts pairs considered is 6,961. The first two columns indicate the species name and whether it belongs to the ZT or the KLE clade. The third column indicates pairs of genes in S. cerevisiae whose orthologs in the parental species are also found together in the genome. Column four indicates the percentage of pairs found conserved. Column number five indicates pairs of genes whose gene order was conserved uniquely between S. cerevisiae and a given parent. The last column indicates the number of pairs whose gene order was conserved exclusively between S. cerevisiae and the ZT-KLE species. (DOCX) [file pbio.1002220.s021.docx]

**S6 Table:** Gene order conservation of pairs of genes between yeast and different species from putative parental clades.

| Group | Species name | Number of conserved pairs | Percentage | Number of uniquely conserved pairs | Number of group conserved pairs |
| --- | --- | --- | --- | --- | --- |
| ZT | *Torulaspora delbrueckii* | 2517 | 44.40% | 69 | 171 |
| ZT | *Zygosaccharomyces rouxii* | 2012 | 35.50% | 17 | 171 |
| KLE | *Ashbya gossypii* | 2217 | 39.10% | 41 | 239 |
| KLE | *Kluyveromyces lactis* | 2258 | 39.90% | 27 | 239 |
| KLE | *Lachancea kluyveri* | 2426 | 42.80% | 7 | 239 |
| KLE | *Lachancea thermotolerans* | 2390 | 42.20% | 14 | 239 |
| KLE | *Lachancea waltii* | 2336 | 41.20% | 15 | 239 |
