# Supplementary material for: Beyond the Whole-Genome Duplication: Phylogenetic Evidence for an Ancient Interspecies Hybridization in the Baker's Yeast Lineage
Source: PLoS Biol. 2015 Aug 7;13(8):e1002220. doi: 10.1371/journal.pbio.1002220 (PMC4529251; doi:10.1371/journal.pbio.1002220)
Supplement: S7 Table — The first two columns indicate the two parents used in the analysis. The third column indicates the number of pairs of genes placed consecutively in a ZT species whose orthologs in a KLE are also placed together in the genome. Column four indicates the percentage of pairs that column three represents. The final column indicates the orthologs in KLE that do not conserve gene order when compared to ZT. (DOCX) [file pbio.1002220.s022.docx]

**S7 Table:** Gene order conservation of pairs of genes between ZT and KLE species.

| ZT | KLE | Number of conserved pairs | Percentage | Orthologous pairs without gene order conservation |
| --- | --- | --- | --- | --- |
| *T. delbrueckii* | *K. lactis* | 3531 | 68.43 | 633 |
| *T. delbrueckii* | *A. gossypii* | 3454 | 66.93 | 471 |
| *T. delbrueckii* | *L. Kluyveri* | 3830 | 74.22 | 543 |
| *T. delbrueckii* | *L. thermotolerans* | 3758 | 72.83 | 476 |
| *T. delbrueckii* | *L. waltii* | 3658 | 70.89 | 501 |
| *Z. rouxii* | *K. lactis* | 3540 | 70.36 | 751 |
| *Z. rouxii* | *A. gossypii* | 3434 | 68.26 | 589 |
| *Z. rouxii* | *L. Kluyveri* | 3854 | 76.61 | 644 |
| *Z. rouxii* | *L. thermotolerans* | 3769 | 74.92 | 578 |
| *Z. rouxii* | *L. waltii* | 3665 | 72.85 | 598 |
