# Supplementary material for: Beyond the Whole-Genome Duplication: Phylogenetic Evidence for an Ancient Interspecies Hybridization in the Baker's Yeast Lineage
Source: PLoS Biol. 2015 Aug 7;13(8):e1002220. doi: 10.1371/journal.pbio.1002220 (PMC4529251; doi:10.1371/journal.pbio.1002220)
Supplement: S8 Table — The first two columns indicate, in this order, species name and data source. The two additional columns represent the number of times the genome was used to reconstruct complete phylomes and reduced phylomes, respectively. Asterisks denote that the species was used as seed. Sources listed: Genolevures: http://www.genolevures.org/; Joint Genome Institute (JGI): http://www.jgi.doe.gov/; National Center for Biotechnology Information (NCBI): http://www.ncbi.nlm.nih.gov/genbank/; Quest for Orthologs: http://questfororthologs.org/; UniProt: http://www.uniprot.org/; and YGOB: http://ygob.ucd.ie/. (DOCX) [file pbio.1002220.s023.docx]

**S8 Table:** List of proteomes used in the main phylomes

| Species name | Source | Number of complete phylomes used | Number of Reduced phylomes used |
| --- | --- | --- | --- |
| *Ashbya gossypii* | Uniprot | 3 | 2 |
| *Candida albicans* | Quest for orthologs | 3 | 18 |
| *Candida castellii* | NCBI | 3 | 0 |
| *Candida glabrata* | Genolevures | 3* | 4* |
| *Debaryomyces hansenii* | Uniprot | 3 | 0 |
| *Dekkera bruxellensis* | JGI | 3 | 0 |
| *Kazachstania africana* | Uniprot | 3 | 0 |
| *Kazachstania naganishii* | YGOB | 3 | 0 |
| *Kluyveromyces lactis* | Uniprot | 3 | 6 |
| *Lachancea kluyveri* | Genolevures | 3 | 6 |
| *Lachancea thermotolerans* | Genolevures | 3 | 2 |
| *Lachancea waltii* | YGOB | 3 | 2 |
| *Nadsonia fulvescens* | JGI | 3 | 0 |
| *Nakaseomyces bacillisporus* | NCBI | 3 | 0 |
| *Naumovozyma castellii* | YGOB | 3 | 0 |
| *Naumovozyma dairenensis* | Uniprot | 3 | 0 |
| *Saccharomyces bayanus* | YGOB | 3 | 0 |
| *Saccharomyces cerevisiae* | Quest for orthologs | 3* | 10* |
| *Scheffersomyces stipitis* | Uniprot | 3 | 0 |
| *Schizosaccharomyces pombe* | Quest for orthologs | 3 | 18 |
| *Tetrapisispora blattae* | YGOB | 3 | 0 |
| *Tetrapisispora phaffii* | Uniprot | 3 | 0 |
| *Torulaspora delbrueckii* | YGOB | 3 | 9 |
| *Vanderwaltozyma polyspora* | YGOB | 3* | 4* |
| *Wickerhamomyces anomalus* | JGI | 3 | 0 |
| *Yarrowia lipolytica* | Genolevures | 3 | 18 |
| *Zygosaccharomyces rouxii* | Genolevures | 3 | 9 |
