# Supplementary material for: Beyond the Whole-Genome Duplication: Phylogenetic Evidence for an Ancient Interspecies Hybridization in the Baker's Yeast Lineage
Source: PLoS Biol. 2015 Aug 7;13(8):e1002220. doi: 10.1371/journal.pbio.1002220 (PMC4529251; doi:10.1371/journal.pbio.1002220)
Supplement: S9 Table — The first two columns indicate, in this order, species name and data source. The third column contains the ID of the phylome a species was used in. See S2 Table for phylomeDB ID correspondence. Asterisks denote the species was used as seed in the phylome. Additional sources listed: Broad Institute: http://www.broadinstitute.org/; Hyphal Tip: http://fungalgenomes.org/blog/available-genomes/; Saccharomyces Genome Database (SGD): http://www.yeastgenome.org/. (DOCX) [file pbio.1002220.s024.docx]

**Table S9:** List of proteomes used in additional phylomes

| Species name | Source | Phylomes used |
| --- | --- | --- |
| *Alternaria Brassicicola* | JGI | 211 |
| *Ashbya gossypii* | Uniprot | 209; 210 |
| *Aspergillus fumigatus* | Uniprot | 211 |
| *Batrachochytrium dendrobatidis* | JGI | 252 |
| *Botrytis fuckeliana* | Broad Institute | 211 |
| *Cochliobolus heterostrophus* | JGI | 211 |
| *Cryptococcus neoformans* | Broad Institute | 252 |
| *Encephalitozoon cuniculi* | Broad Institute | 252 |
| *Homoloaphlyctis polyrhiza* | JGI | 252 |
| *Hortaea werneckii* | NCBI + annotation | 211* |
| *Kluyveromyces lactis* | Uniprot | 209; 210 |
| *Lichtheimia corymbifera* | Sequencing project | 252 |
| *Macrophomina phaseolina* | Uniprot | 211 |
| *Monosiga brevicollis* | JGI | 252 |
| *Mortierella alpina* | NCBI + annotation | 252 |
| *Mucor circinelloides* | JGI | 252 |
| *Mycosphaerella fijiensis* | JGI | 211 |
| *Naumovozyma castellii* | YGOB | 209; 210 |
| *Nematocida parisii* | Broad Institute | 252 |
| *Nematostella vectensis* | JGI | 252 |
| *Neurospora crassa* | Uniprot | 252 |
| *Nosema ceranae* | Broad Institute | 252 |
| *Phaeosphaeria nodorum* | Quest for orthologs | 211 |
| *Phycomyces blakesleeanus* | JGI | 252 |
| *Puccinia graminis* | JGI | 252 |
| *Rhizopus oryzae* | Broad Institute | 252* |
| *Rhytidhysteron rufulum* | JGI | 211 |
| *Saccharomyces bayanus* | YGOB | 209; 210 |
| *Saccharomyces cerevisiae* | Quest for orthologs | 209; 210 |
| *Saccharomyces cerevisiae x Saccharomyces kudriavzevii VIN7* | NCBI | 210* |
| *Saccharomyces kudriavzevii* | Hyphal tip | 209; 210 |
| *Saccharomyces mikatae* | SGD | 209; 210 |
| *Saccharomyces paradoxus* | SGD | 209; 210 |
| *Saccharomyces pastorianus* | NCBI + annotation | 209* |
| *Schizosaccharomyces pombe* | Broad Institute | 252 |
| *Ustilago maydis* | Quest for orthologs | 252 |
| *Zymoseptoria tritici* | Uniprot | 211 |
